# Reactive astrogliosis in response to hemorrhagic fever virus: microarray profile of Junin virus-infected human astrocytes

**DOI:** 10.1186/1743-422X-11-126

**Published:** 2014-07-11

**Authors:** Olga A Kolokoltsova, Nadezhda E Yun, Slobodan Paessler

**Affiliations:** 1 Department of Pathology, Galveston National Laboratory, University of Texas Medical Branch, 301 University Boulevard, Galveston, TX, USA

**Keywords:** Interferon, Apoptosis, Inflammation, Neurodegeneration

## Abstract

**Background:**

Arenavirus Junin is the causative agent of Argentine hemorrhagic fever. Limited information is available concerning the pathogenesis of this human disease, especially the pathogenesis of acute and late neurological symptoms.

**Methods:**

In our study we present for the first time cDNA microarray profile of human astrocytes infected with the virulent strain of Junin virus. Transcriptional profiling was confirmed by quantitative real-time RT-PCR and cytokine/chemokine/growth factor assay.

**Results:**

We demonstrated the impact of virus infection on immune/inflammatory response/interferon signaling and apoptosis. Pro-apoptotic response and amplification with time of pro-inflammatory cascade of human astrocytes suggested neurodegenerative dysfunctional reactive astrogliosis in response to Junin virus infection.

**Conclusion:**

Our results suggest potential pathogenic role of astroglial cells in the development of neurological symptoms and late neurological syndrome during Argentine hemorrhagic fever.

## Introduction

New World clade B arenavirus Junin [1] is the etiological agent of Argentine hemorrhagic fever (AHF) [2], highly lethal disease endemic to the central Argentina [3]. Inhalation of aerosolized rodent secreta and excreta or direct contact with infected rodents of Calomys musculinus and laucha spp, chiefly drylands vesper mouse, are the main routes of infections among agricultural workers during harvest season [3,4]. The disease case fatality rate is 15-42% when untreated [5,6]. The highest level of biocontainment (BSL-4) is required for the work with most of the hemorrhagic fever viruses in the USA, including JUNV, which hinders biological research in this field.

AHF is characterized by gastrointestinal, cardiovascular, hematological, renal, immunological, hemorrhagic and neurological manifestations [5,7]. As a matter of fact, many patients infected with various hemorrhagic fever viruses present with either acute or chronic neurological disorders. The pathogenesis of the disease remains elusive. Elevated levels of IL-6, IL-8, IL-10 and TNF-α detected in AHF patients correlated with the disease severity [8,9]; while increased concentration of G-CSF in serum of AHF patients correlated with levels of TNF-α, IL-6, IL-8, and IL-10 [10]. In addition, elevated levels of IFN-α detected in patients with AHF correlated with severity of disease, poor prognosis [11], fever, chills, backache [12], low platelet count and platelet abnormality [13]. The source of these cytokines and chemokines in response to JUNV infection in humans is unclear. No induction of IFN-α, IFN-β, IL-6, IL-10, IL-12, or TNF-α production was observed in *ex vivo* cultivated human monocytes and macrophages upon infection with pathogenic Romero strain of JUNV [14]. In contrast, recently we demonstrated type I interferon (IFN) production, IFN stimulated gene expression and STAT1 phosphorylation in JUNV Romero-infected human lung epithelial carcinoma cells. Additionally, we showed that in these cells RIG-I/IRF3 signaling was responsible for type I IFN induction upon JUNV infection [15].

Neurologic signs are relatively common during AHF (10% of symptomatic case) [16], however underlying pathological changes are not understood. Although, JUNV was isolated from brain tissues obtained during autopsy of fatal cases of AHF [17], no neuronal necrosis was observed [16]. Histopathological findings in the CNS of patients with neurological cases of AHF also include severe meningeal congestion, hemorrhage in Virchow Robin space, lymphocytic perivascular infiltrates in the brain and meninges [17,18], diffuse microglial proliferation [17] and capillary lesions [16]. Moreover, a study of 10 autopsy cases of AHF described focal and diffused glial cell proliferation and edema in all patients, and brain microhemorrhages in some. In contrast to the aforementioned autopsy report [16], chromatolysis and pyknosis in neurons suggestive of neuronal apoptosis and/or necrosis was detected in this study [19].

JUNV is increasingly neurotropic in the most relevant primate models of AHF [20-23]. Infectious virus was found in the brains of New World primates *Callithrix jacchus* intramuscularly infected with JUNV XJ strain. Brain pathology of the infected animals included lymphoreticular perivascular cuffing, gliosis, and leptomeningitis. Viral antigen was detected in the brain via immunostaining in the small vessels endothelium, neurons and leptomeninges [20].

A late neurological syndrome (LNS) develops in 11% of AHF survivors treated with convalescent serum [24,25]. No human pathology data exist to shed light on the mechanism of this complication. Modeling of LNS in the guinea pig model provided some insights to the disease development. Following intraperitoneal infection with Romero JUNV, untreated guinea pigs succumbed to infection after 2 weeks with no apparent brain pathology. In contrast, animals treated with JUNV-specific immune sera developed hind leg paralyses 3 weeks post challenge. High titer of infectious virus was found in the brain but not in the other organs of those animals. Histopathological findings included swollen vascular endothelium, encephalitic and meningeal perivascular cuffs made of lymphocytes and monocytes as well as infiltrates of macrophages and swollen astrocytes indicative of neuronal degeneration [26]. Similarly, JUNV XJ strain-infected immune serum treated non-human primates *Callithrix jacchus* develop neurologic complications including hind-limb paralysis. High titer of infectious virus was found in the brain and lesions consisting of perivascular mononuclear infiltrates and neuronal necrosis were detected in the spinal cord of the affected animals [27].

Since the pathogenesis of AHF is not clearly understood, we utilized cDNA microarray technology to profile *in vitro* transcriptional changes associated with JUNV infection of human cells. Published pathology findings from both humans with AHF and the relevant animal models suggest that glial cells, particularly astrocytes, may play an important role in the neurological disorders associated with AHF. Accordingly, we have selected normal human astrocytes (NHA), non-transformed, non-immortalized, primary cells for transcriptional profiling upon infection with the virulent strain of JUNV, Romero.

## Materials and methods

### Virus

The Romero strain of JUNV (GenBank accession nos. AY619640 and AY619641) was obtained from Dr. Thomas G. Ksiazek (Centers for Disease Control and Prevention, Atlanta, GA). The virus was isolated from a patient and passaged twice in fetal rhesus lung cells and once in Vero cells [28]. Laboratory virus stock was obtained by amplification in Vero E6 cells. Cell debris in supernatants were filtered out through 0.45 μm HV Durapore Membrane Stericup sterile vacuum filtration system (Millipore Corporation, Billerica, MA). Cleared supernatants were concentrated through 30 min centrifugation at 3220 × g using Amicon Ultra-15 Centrifugal Filter Unit PLHK Ultracel-PL Membrane, 100 kDa (Millipore Corporation, Billerica, MA). All work with JUNV was performed at the University of Texas Medical Branch BSL-4 facilities (Robert E. Shope Laboratory) in accordance with institutional health and safety guidelines and federal regulations [29].

### Cell lines

Clonetics Normal Human Astrocytes cells were maintained in ABM Astrocyte Basal Medium supplemented for a complete growth with AGM SingleQuots (Lonza Walkersville, Inc., Walkersville, MD). Vero cells (American Tissue Culture Collection, Manassas, VA) were maintained in minimum essential medium (MEM) supplemented with 10% fetal bovine serum and 1% Penicillin-Streptomycin (10,000 U/mL).

### JUNV replication in NHA

NHA from a single donor were seeded at a concentration of 5 × 10^5^/35-mm dish. After a 4-h incubation at 37°C monolayers were infected at a multiplicity of infection (MOI) = 0.01, 0.1 or 1 PFU/cell and incubated for 1 h at 37°C. Fresh medium was used for mock infection. The inoculum was then replaced with growth medium. At selected times after infection, supernatants were harvested, and virus titers determined by a plaque assay on Vero cells as previously described [30]. Data were analyzed by two-way ANOVA using SigmaPlot 12.0 (Systat Software, Inc., San Jose, CA).

### Total RNA preparation and GeneChip processing

NHA were seeded at 7.5 × 10^5^ cells/60 mm dish in 4 mL of growth medium. After 24 h, medium was removed and cells were mock-infected or infected with JUNV Romero for 1 h at an MOI = 5 in 0.5 mL of medium. Then, medium was replaced for incubation of cells.

DNase-treated total RNA was isolated from cells using TRIZOL Reagent (Invitrogen Corporation, Carlsbad, CA) followed by the RNAqueous Mini and RNeasy-Free DNAase Set (QIAGEN Inc., Valencia, CA) according to the protocols adapted from the manufacturer’s instructions. Total RNA (0.5 μg) was subjected to GeneChip Expression Analysis (Affymetrix, Santa Clara, CA) according to the manufacturer’s instructions [Expression Analysis Technical Manual, Section 2: Eukaryotic Sample and Array Processing, Alternative protocol for One-Cycle cDNA synthesis followed by synthesis of biotin-labeled cRNA with MessageAmp Premier RNA Amplification Kit (Ambion Inc, Austin, TX)] in the Molecular Genomics Core (University of Texas Medical Branch, Galveston,TX). Total fragmented cRNA (10 μg) was hybridized to the Affymetrix Human Genome GeneChip array U133A 2.0 using the GeneChip Hybridization Oven 640. The chips were washed and stained in a GeneChip Fluidics Station 450 and fluorescence was detected with an Affymetrix-GS3000 Gene Array scanner using the GeneChip Operating System software (GCOS1.4).

### Microarray analysis

Microarrays quality assessment, preprocessing and differential expression analysis were performed with Bioconductor software packages [31] in R programming environment [32]. Microarray quality was assessed with affy [33], affyPLM [34] and QCReport packages. Log2 scale expression measures were generated using the gcRMA algorithm of preprocessing [35] using the gcrma package. General similarity in expression patterns of preprocessed microarrays was assessed by principal component analysis using the affycoretools package. The detection calls (present, marginal, or absent) for each probe set were obtained using the mas5calls function in the affy package [33]. Only genes with at least one present call across all samples were included.

Differential gene expression was analyzed using limma package by fitting a linear model to the data to estimate the variability and by using an empirical Bayesian method to moderate the standard errors of the estimated log-fold changes for statistical analysis and assessing differential expression [36]. The moderated t-statistic associated P-values derived from the linear-model analysis were adjusted for multiple testing according to Benjamini and Hochberg’s method to control the false discovery rate (FDR) [37]. The smallest adjusted P-value among control and spike-in probe sets, which should not be differentially expressed, was used to define P-value cutoffs. The FDR (setting the P-value < 0.0005 avoided selecting any of the control probe sets as differentially expressed) and a log2-fold change > 1 was used to obtain the list of differentially expressed genes.

Hierarchical clustering was performed on a set of differentially expressed genes using the Spotfire DecisionSite 9.0 for Functional Genomics (Spotfire Inc., Somerville MA). The pairwise clustering algorithm employed the Unweighted Pair-Group Method with Arithmetic Mean method and defined the similarity between clusters by their Euclidian distance [38]. Both probe sets (rows) and chips (columns) were blindly clustered, with the final order determined by average values and not by input order.

Gene functionality of differentially expressed genes was analyzed using the functional analysis tool of the Ingenuity Pathways Analysis (Ingenuity Systems, http://www.ingenuity.com). Differentially expressed genes were associated with biological functions and/or diseases in Ingenuity’s Knowledge Base. Right-tailed Fisher’s exact test was used to calculate a P-value determining the probability that each biological function and/or disease assigned to that data set is due to chance.

Differentially expressed transcripts were associated with canonical pathways that were most significant to the data set in Ingenuity Knowledge Base. The significance of the association between the data set and the canonical pathway was measured in two ways: A ratio of the number of molecules from the data set that map to the pathway divided by the total number of molecules that map to the canonical pathway displayed. Fisher’s exact test was used to calculate a P-value determining the probability that the association between the genes in the dataset and the canonical pathway is caused by chance.

To identify an effect of transcriptional changes on biological functions Ingenuity Pathways Analysis Downstream Effects analytic was implemented. The downstream effects analysis was based on prior knowledge of expected causal effects between genes and biological functions stored in the Ingenuity Knowledge Base. The increase/decrease of biological function in response to detected transcriptional changes was concluded based on the activation z-score and P-value calculated with the Fischer’s exact test.

To identify the upstream transcriptional regulators responsible for the observed gene expression changes the Ingenuity Pathways Upstream Regulator Analysis was used. The analysis was based on prior knowledge of expected effects between transcriptional regulators and their target genes stored in the Ingenuity Knowledge Base. The analysis examined the number of each transcription regulator targets and their direction of change to what is expected from the literature. The upstream transcriptional regulators were selected based on the activation z-score and P-value calculated with the Fischer’s exact test.

### Quantitative real-time RT-PCR (qRT-PCR)

cDNA synthesis and PCR amplification were performed with iScript cDNA Synthesis Kit and iQ SYBR Green Supermix (Bio-Rad, Hercules, CA) according to the manufacturers’ instructions. Real-time PCR was performed on CFX96 Real-Time PCR Detection System (Bio-Rad, Hercules, CA). The “Baseline Subtracted Curve Fit” analysis mode with user defined baseline and threshold values was used to determine the geometric mean of the threshold cycle (Ct) using CFX Manager Software Version 1.5 (Bio-Rad, Hercules, CA). To control for variation in input RNA quantities and reverse transcription efficiencies, Ct values were normalized to the average Ct values of glyceraldehyde-3-phosphate dehydrogenase (GAPDH) and actine β (ACTB) housekeeping genes. Melt curve analysis of the PCR products was used to confirm specificity. Primers were designed from target mRNA sequences obtained through NetAffx Analysis Center (Affymetrix, Santa Clara, CA) using NCBI/Primer-BLAST software [39]. The qRT-PCR primer pair sequences will be supplied upon request. ΔΔCt based fold-change calculations and statistical analysis (Student’s t-test) of quantitative real-time RT-PCR data were performed at the RT^2^ Profiler™ PCR Array Data Analysis Web Portal (http://www.sabiosciences.com/pcr/arrayanalysis.php).

### Quantification of cytokines, chemokines, and growth factors in supernatants

NHA astrocytes were plated and infected as described in Total RNA preparation and GeneChip processing. Supernatants were harvested at 0, 24 and 96 h p.i. and processed using Bio-Plex Pro™ human cytokine 27-plex immunoassay (Bio-Rad, Hercules, CA) according to the manufacturers’ instructions. Concentration of the following factors were assayed: basic FGF, G-CSF, GM-CSF, PDGF-BB, VEGF, IL-1β, IL-1ra, IL-2, IL-6, IL-7, IL-8, IL-9, IL-15, IL-12 (p70), IL-4, IL-5, IL-10, IL-13, IL-17, eotaxin, IP-10, MCP-1 (MCAF), MIP-1α, MIP-1β, RANTES, IFN-γ, TNF-α.

### Quantification of IFN-β in supernatants

Next day after plating 1 × 10^4^ NHA cells were mock-treated or treated with ranging concentrations of Poly(I:C)-LMW/LyoVec (InvivoGen, San Diego, CA) per manufacturers’ instructions. Next day after plating 1 × 10^4^ NHA cells were mock-infected or infected with VEE TC-83 virus for 1 h at an MOI = 0.1 PFU/cell. At 24 h posttreatment or post infection (p.i.) cells supernatants were collected and concentration of human IFN-β was assayed by sandwich ELISA method using VeriKineTM Human IFN-Beta ELISA Kit (PBL Assay Science, Piscataway, NJ) according to the manufacturer’s instruction. Assay calibration limit is 50 pg/mL.

## Results

### JUNV infects NHA

**Figure 1:**
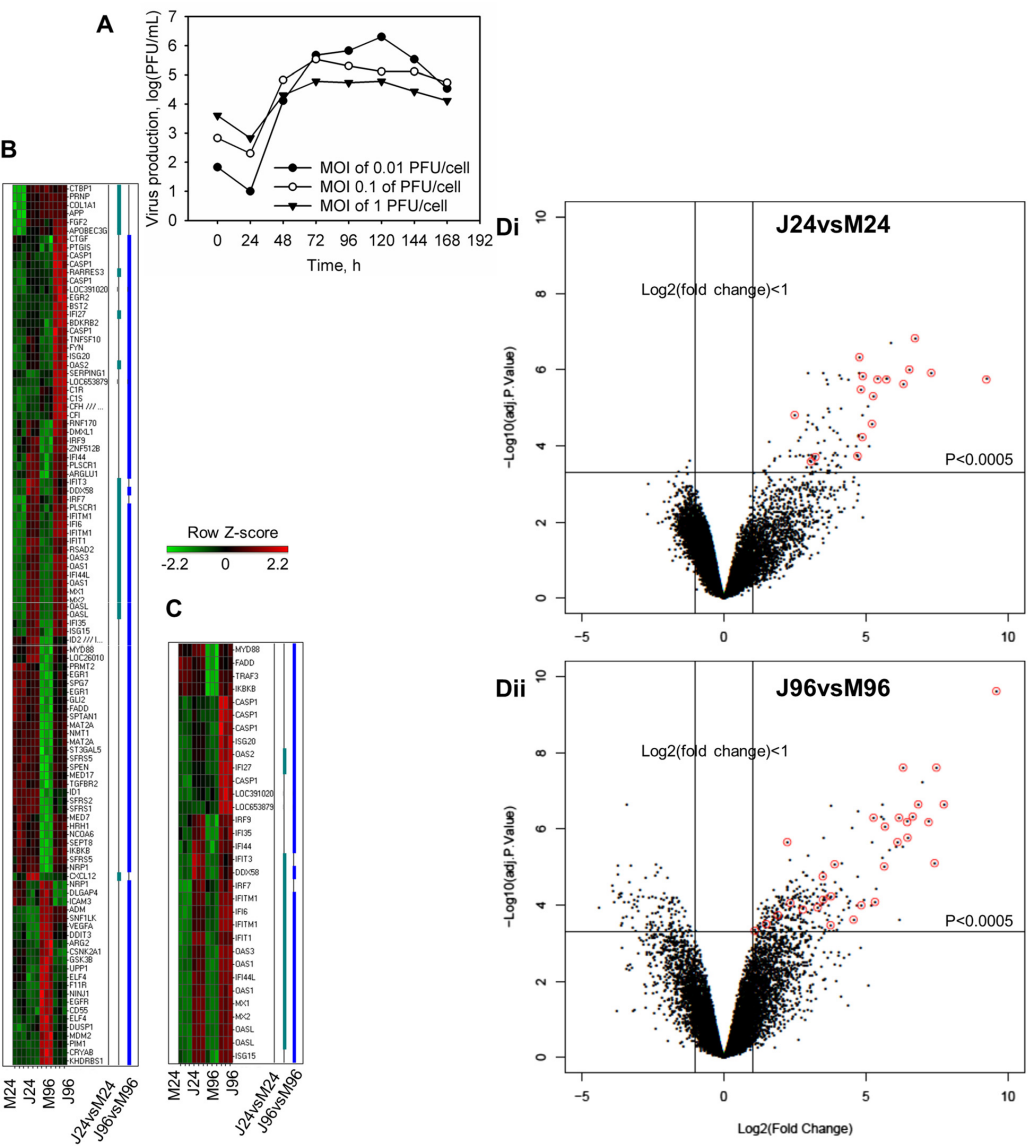
**JUNV productive infection of NHA leads to amplification with time of immune/inflammatory response. A**. JUNV production in NHA. Cells were infected with the virus at indicated MOIs and supernatants were harvested daily. Production of infectious virus was measured by plaque assay. **B**, **C**. Heat map plot of differentially expressed Immune/Inflammatory **(B)** and IFN **(C)** signaling transcripts for 24 and 96 h p.i. Rows are arranged by hierarchical clustering following Z-score transformation of the normalized data. Columns represent mock-infected (M24 and M96) and JUNV-infected (J24 and J96) tissue culture triplicates at 24 and 96 h p.i., respectively. Blue and green notches represent differentially expressed IFN signaling transcripts for 24 and 96 h time points p.i., respectively. **D**. Volcano plots of microarray results with identification of differentially expressed IFN-stimulated genes transcripts (red circles) for 24 **(Di)** and 96 **(Dii)** h p.i. The horizontal line represents significance (P < 0.0005) and the vertical lines represent the change in gene expression of JUNV-infected NHA compared with that of Mock-infected cells [Log2(fold change) > 1].

To determine the susceptibility and potential kinetics of JUNV replication in astrocytes, we infected NHA cells at a multiplicity of infection (MOI) of 0.01, 0.1 and 1.0 PFU/cell, and then collected virus supernatants for virus titration every 24 h over a period of 6 days (Figure 1A). No production of infectious virus was detected during the first 24 h p.i. Production rose exponentially during the following 72 h, peaking 96 h p.i. Levels of JUNV (Romero strain) were slightly higher (although statistically insignificant) for the lower initial MOI; however, production kinetics were similar among the different inoculations. These data indicate that JUNV is capable of infecting NHA, resulting in high yields of infectious virus.

### Microarray analysis of NHA infected with JUNV

To gain insight into the response of human glial cells to JUNV infection, we performed a microarray study of the transcriptional changes that occur in infected and mock-infected NHA cells. Tissue culture triplicates of NHA were infected or mock-infected with JUNV at an MOI of 5 PFU/cell. Based on the kinetics of JUNV replication in NHA, two time points were chosen for microarray analysis. The first time point at 24 h p.i. represented an early time point associated with establishment of infection, while the second time point at 96 h p.i. allowed us to profile transcriptional changes related to JUNV infection after overcoming host defenses. Furthermore, the transcriptional state of cells can be affected by cellular proliferation or by reaching confluency. In order to minimize the confounding effects of time-associated changes, we compared gene expression data from the infected samples at each time point with the corresponding mock-infected control.

At 24 and 96 h p.i., total RNA was isolated from NHA cells infected with JUNV or mock-infected and was subjected to GeneChip expression analysis. A list of differentially expressed genes, which possess low probability values adjusted for multiple testing (P < 0.0005) and log2-fold changes of more than 1, was obtained as described in the Materials and Methods. At 24 h p.i., 3 transcripts were more than 2-fold down-regulated and 92 transcripts were more than 2-fold up-regulated in the JUNV-infected NHA, compared with mock-infected controls. At the 96 h p.i. time point, a total of 112 differentially expressed genes were down-regulated and 266 were up-regulated. Of these, 21 transcripts were up-regulated at both time points. The number of up-regulated, differentially expressed transcripts in JUNV-infected cells was higher than that of down-regulated genes at both time points.

The entire dataset of differentially expressed genes was further subjected to functional analysis through association with biological function/disease and canonical pathways. An effect of transcriptional changes on biological functions as well as the upstream transcriptional regulators responsible for the observed gene expression changes were identified. Genes that were differentially expressed in the JUNV-infected NHA compared to mock-infected controls belonged to many different gene categories, including those involving the host immune response, apoptosis and cell cycle. Apoptosis genes and immune response genes, especially IFN-stimulated genes, stood out with respect to consistency of differential expression, number of differentially expressed genes, and overall change.

### Amplification of immune/inflammatory response during JUNV infection

Genes regulated upon infection with JUNV were grouped based on functional annotations. Over 23% (105 of total 452) of differentially expressed genes were categorized as participating in several functionally overlapping categories, which included antigen presentation, antimicrobial response, cell-mediated immune response, humoral immune response, inflammatory response, organismal injury and abnormalities, infection mechanism and infection disease. We collectively named these categories the immune/inflammatory response. We found 27 (out of 95 total) and 96 (out of 378) differentially expressed transcripts that were classified as involved in the immune response at both 24 and 96 h p.i., indicating increased cellular immune response following JUNV infection (Figure 1B). Interestingly, at 24 h p.i., all immune response-related transcripts were up-regulated. At 96 h p.i., the majority of inflammatory response transcripts were still up-regulated; however, 22 out of 96 transcripts were down-regulated (Figure 1B).Of all immune response transcripts, 17 (out of the 27 total) at 24 h p.i., and 30 (out of the 96 total) at 96 h p.i. could be categorized as related to the IFN signaling transduction (JAK-STAT pathway) or induction [pattern recognition receptors (PRR) involved in the recognition of bacteria and viruses, RIG-I-like receptors involved in antiviral innate immunity and the activation of IRF by cytosolic PRRs canonical pathways] (Figure 1C). Quite strikingly, all IFN signaling transcripts were up-regulated at both time points p.i. (Figure 1C, D). The most highly up-regulated transcripts could almost all be categorized as related to the IFN signaling (Figure 1D).

**Figure 2:**
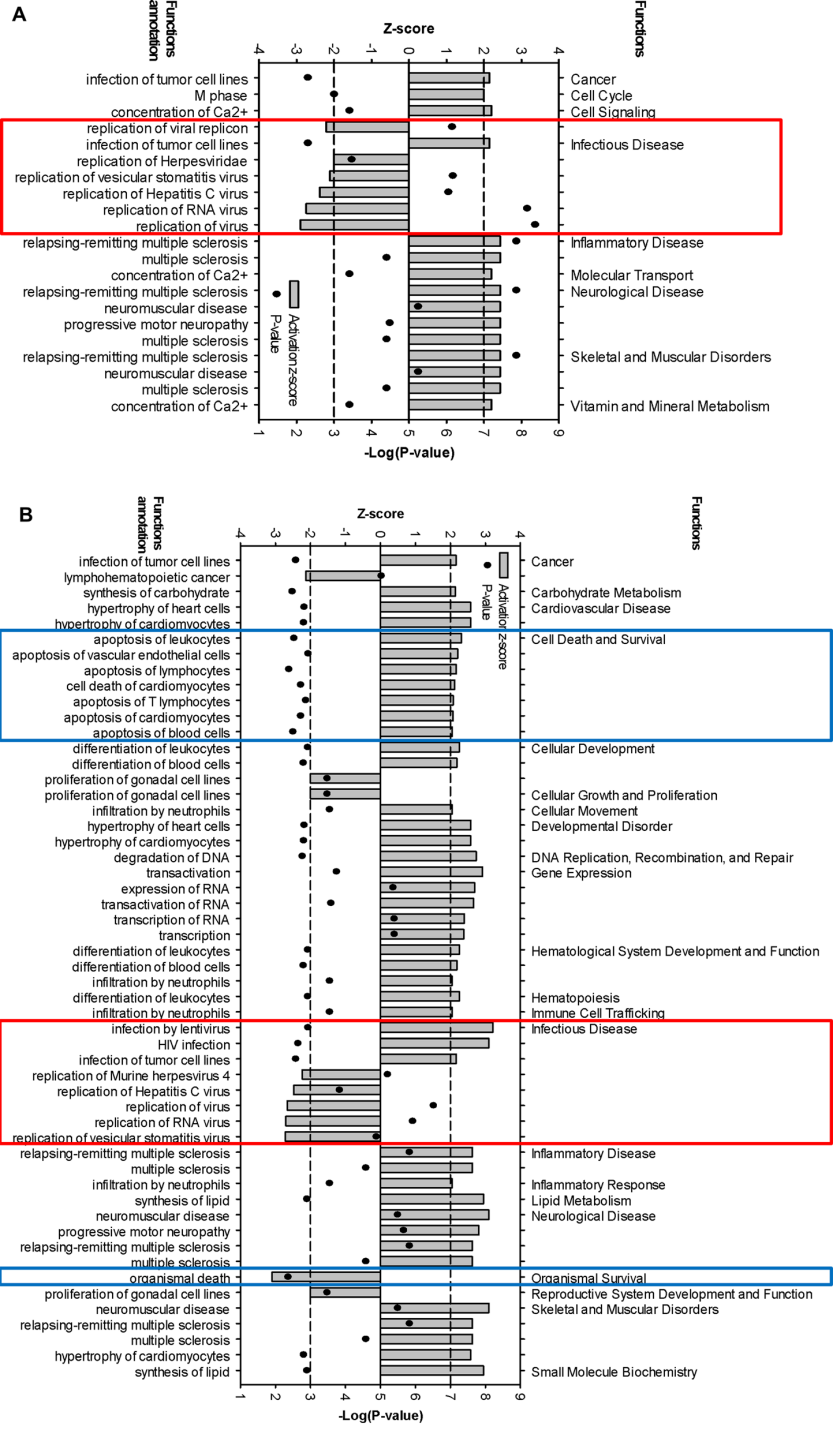
**Predicted increase or decrease of biological functions in NHA in response to JUNV infection at 24 (A) and 96 (B) h p.i.** Significance of Downstream Effects analysis was based on combination of the activation z-score (-2 ≥ Z ≥ 2) and overlap P-value (P < 0.05). The horizontal dashed lines represent activation z-score significance cutoff. Red and blue boxes highlight Infection Diseases and Cell Death functions, respectively.

Differentially expressed IFN-stimulated transcripts included those known to be up-regulated in response to type I IFNs (IFIT1, IFIT3, MX1 and OAS) or associated with type I induction (IRF7 and IRF9) (Figure 1C). In addition, both Downstream Effects (Figure 2) and Upstream Regulator (Additional file 1: Table S1) analyses confirmed an induction of immune/inflammatory response and, particularly, IFN signaling upon Romero JUNV infection. In agreement with type I IFN signaling induction, biological function related to viral replication were decreased while Inflammatory and Neurological disease functions were uniformly increased in Romero-infected NHA (Figure 2) at 24 and 96 h p.i. For complete list of differentially expressed gene for each function see Additional file 2: Table S2 and Additional file 3: Table S3. Although no type I IFN transcripts were up-regulated in Romero-infected NHA (Figure 1B, C), IFNs α, β, λ and γ, IFN type I receptor IFNAR1 and key transcriptional factors in induction of type I IFN signaling including IRF-7, STAT1 and STAT2 were predicted to be activated (activation z-score ≥ 2 and overlap P-value < 0.05) at both time points p.i. (Additional file 1: Table S1).

**Figure 3:**
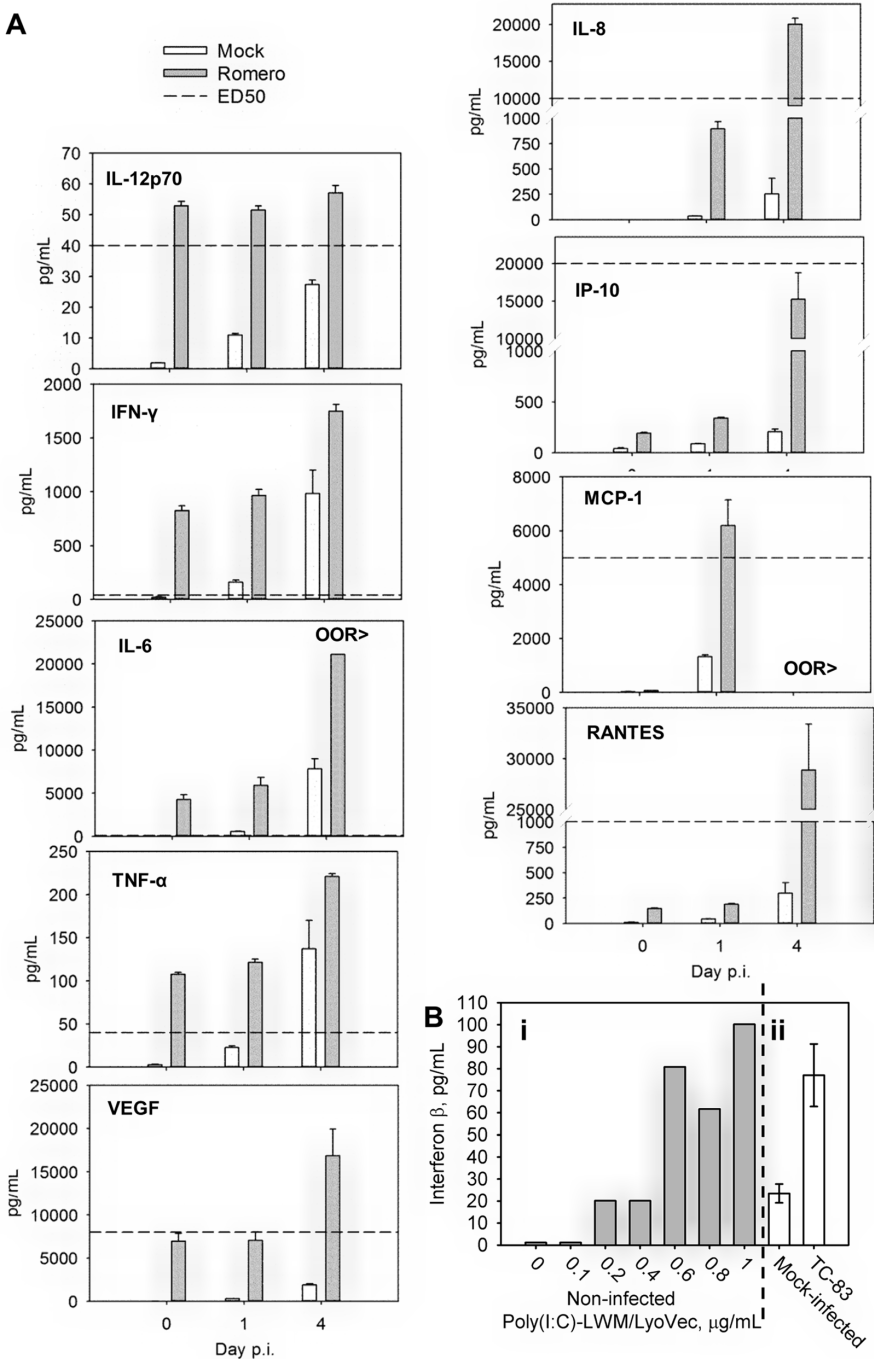
**Cytokines, chemokines, and growth factors production in NHA. A.** Cytokines, chemokines, and growth factors production in NHA in response to JUNV infection. Cells were mock-infected or infected with the virus and supernatants were harvested at 0, 24 and 96 h p.i.. Quantification of cytokines, chemokines, and growth factors was performed by Bio-Plex multiplex assay. OOR>, out of standard range, above. Data represent the average of 3 replicates ± SEM. **B.** IFN-β induction in NHA upon stimulation with poly(I:C) and vaccine strain of VEE virus. Cells supernatants were assayed using human IFN-β ELISA. Bi. Cells were treated with ranging concentrations of Poly(I:C)-LMW/LyoVec or mock-treated. Bii. Cells were mock-infected or infected with VEE TC-83 virus. Data represent the average of 3 technical replicates ± SD.

In agreement with the role of RIG-I/IRF3 signaling in type I IFN induction in response to JUNV infection [15], an expression of RIG-I (DDX58) and IRF3-dependent genes [40] [IFIT1 (ISG56); IFIT3 (ISG60, up-regulated at 24 h p.i.); ISG-15 (up-regulated at 96 h p.i.) were increased in the infected cells (Figure 1C). Moreover, Upstream Regulator analysis predicted an activation of RLH adapter protein MAVS and key transcriptional factors (IRF-1,3,5) involved into PRR-mediated induction of type I IFN at both time points p.i. (Additional file 1: Table S1). By utilizing Bio-Plex multiplex system for the quantification of cytokines, chemokines, and growth factors we showed that production of RANTES, a product of another IRF3-regulated gene [41], was elevated in supernatants collected from Romero-infected NHA. Consistent with PRR-mediated NF-κB signaling activation, we detected an increased production of type I cytokines (IL-12, INF-γ), proinflammatory cytokines (IL-6, TNF-α), chemoattractants and chemokines (IL-8, IP-10, MCP-1) and growth factors (VEGF) in Romero-infected NHA (Figure 3A).

Intriguingly, an expression of MYD88, a universal TLR adaptor, was elevated in Romero JUNV-infected cells at 96 h p.i. (Figure 1C) and an activation status was assigned to TLR3 and its adaptor protein TRIF at both times p.i. and TLR4 at 96 h p.i. (Additional file 1: Table S1).Although INF-γ was produced by JUNV-infected NHA and multiple differentially expressed transcripts simultaneously linked to type I and II IFN response (e.g., IFI27, IFI44, IFI44L, IFI6, IFITM1, ISG20, MX2 and IFI35) were up-regulated in those cells, none of the transcripts specific to type II IFNs were identified in our screen (Figure 1C). These data suggested that type-I IFNs are the main antiviral signaling in human glial cells in response to JUNV infection.

### Validation of microarray data by qRT-PCR and ELISA

Selected genes from the microarray analysis were subjected to validation by qRT-PCR to establish the extent to which gene expression was modulated. Real-time PCR was carried out using cDNA from JUNV-infected NHA and from mock-infected control cells. Modulation of the specific mRNA for 20 genes relative to the average expression of GAPDH and ACTB housekeeping genes was assessed at both times p.i. (Additional file 4: Table S4). The microarray data highly correlated with those obtained by quantitative real-time RT-PCR (Additional file 4: Table S4). The magnitude of upregulation was comparable between the two methods, but was sometimes more extreme in qRT-PCR than in microarray, possibly because quantitative real-time PCR data are more accurate over a broader range.

None of the transcripts representing type I IFNs was detected as differentially expressed by microarray analysis. To account for possibility of type II error we independently validated an expression level of IFN-β (IFNB1), immediate early-phase (IFN-α4) and an autocrine activated (IFN-α2) α-IFNs at both time points p.i. The levels of IFN-α were not significantly different between JUNV-infected and corresponding mock-infected samples as detected by qRT-PCR (data not shown). Interestingly, using the real-time PCR we found an elevated (above mock-infected) level of expression of IFNB1 transcripts in JUNV-infected samples: 17.02 fold (95% CI 0.00001, 39.75; P-value 0.115666) at 24 h and 7.46 fold (95% CI 2.07, 12.85; P-value 0.033561) at 96 h p.i. However, an IFNB1 amplification product was undetectable in mock-infected samples (average Ct values >40) and PCR product was barely detected for infected samples (average Ct values of 36.73 and 36.47 at 24 and 96 h p.i. correspondingly) (data not shown). The following indicates a low abundance of IFNB1-coding RNA transcripts during JUNV infection, considering that the initial load of cDNA was the maximum allowed by the assay. In agreement with low abundance of IFNB1 transcripts, no elevation of IFN-β levels above the background was detected by ELISA in the supernatant of the infected and mock-infected samples at both time points (data not shown). As detected by qRT-PCR, increased levels of IFNB1 transcripts upon infection correlates with induction of IFN-stimulated signaling registered in the microarray analysis. However, the low total abundance of the transcript and secreted protein level probably suggest that the initial peak of IFN-β induction happened before 24 h p.i.Additionally, we validated the integrity of type I IFN signaling pathway in NHA by examining IFN-β production through ELISA in these cells upon PRR stimulation. In contrast to mock-treated cells, NHA showed a robust IFN-β production upon poly(I:C) stimulation (Figure 3Bi) or after infection with the TC-83 vaccine strain of Venezuelan equine encephalitis virus (Figure 3Bii). These data clearly confirm the unimpaired ability of NHA to mount type I IFN response, however overall IFN-β production was low (up to 100 pg/mL of medium).

### Pro-apoptotic response of NHA to JUNV infection

**Figure 4:**
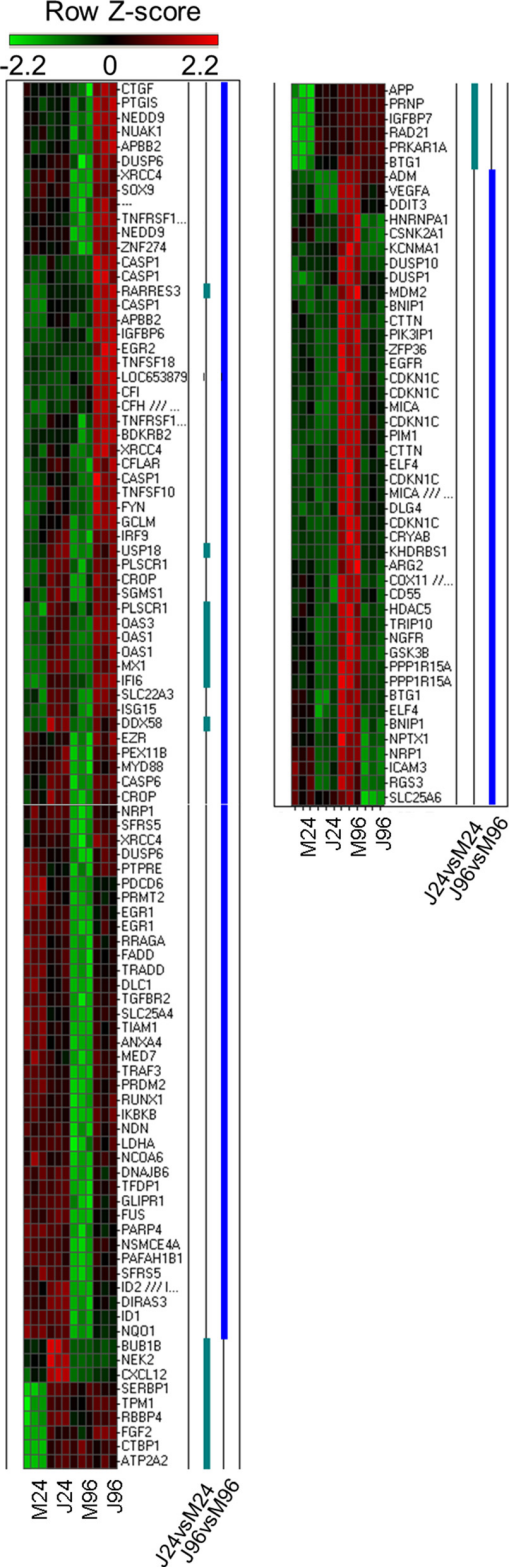
**Differential expression profile of transcripts categorized as regulators of cell death of NHA infected with JUNV.** Heat map plot of differentially expressed cell death associated transcripts for 24 and 96 h p.i. Rows are arranged by hierarchical clustering following Z-score transformation of the normalized data. Columns represent mock-infected (M24 and M96) and JUNV-infected (J24 and J96) tissue culture triplicates at 24 and 96 h p.i., respectively. Blue and green notches represent differentially expressed cell death associated transcripts for 24 and 96 h p.i. respectively.

Cell death genes comprised the other major category of differentially expressed transcripts in JUNV-infected NHA at 96 h p.i. Ingenuity Pathway functional analysis identified cell death as the most significant (P-value <10^6^) functional group for the 96 h p.i., with over 34% (131 of total 378) of differentially expressed transcripts implicated in the complement system: induction of apoptosis by HIV; retinoic acid mediated apoptotic signaling; death receptor signaling; p38 MAPK signaling; NF-κB signaling; and acute phase response signaling canonical pathways (listed in the P-value increasing order). According to the analysis, in the Romero-infected cells, in the Cell Death and Survival functional category, apoptosis of leukocytes, vascular endothelial cells, lymphocytes, T lymphocytes, cardiomyocytes and blood cells was uniformly increased (Figure 2, Additional file 2: Table S2 and Additional file 3: Table S3). Among differentially expressed cell death related genes, CASP6, CFLAR (FLIP), FADD, IKBKB, TNFSF10 (TRAIL), and TRADD transcripts were uniformly up-regulated at 96 h p.i. suggesting the importance of classical Death Receptor Signaling pathway for apoptosis induction JUNV pathogenesis (Figure 4). Additionally, at 96 h p.i. we found 7 differentially expressed transcripts/genes (C3, C1R, C1S, CD55, CFH, CFI and SERPING1) identified as part of the canonical pathway of complement activation. Supportive of a proapoptotic phenotype, expression of 6 of these transcripts was up-regulated (C3, C1R, C1S, CFH, CFI and SERPING1). This observation is consistent with complement system activation for the clearance of apoptotic cells in the brain [42] (Figure 4).

In summary, gene transcriptional profile of JUNV-infected NHA at 96 h p.i. shows a differential expression of genes associated with programmed cell death. Ingenuity Pathway Functional and Downstream Effects analyses suggested an induction of programmed cell death with obvious directionality toward a pro-apoptotic phenotype in the JUNV-infected NHA compared with the mock-infected cells.

## Discussion

We studied infection of NHA with the virulent strain of JUNV and showed that human astrocytes are highly susceptible to infection. Susceptibility of NHA to JUNV infection indicates the possibility that astrocytes might be a source of virus production in the brain of patients with AHF. Additionally, we analyzed the transcriptional response of NHA to JUNV infection early and late post challenge. To our knowledge, this is the first report of JUNV-induced response in human astrocytes, and the first study using mRNA profiling upon infection of human cells. Using transcriptional profiling that was partly confirmed by qRT-PCR and cytokine/chemokine/growth factor assay, we demonstrated the impact of virus infection on both immune/inflammatory response/IFN signaling and apoptosis. Amplification of pro-inflammatory cascade with time and pro-apoptotic response of NHA to JUNV infection are indicative of neurodegenerative dysfunctional reactive astrogliosis [43,44]. In response to neurological injury initial early astrocyte activation and reactive astrogliosis (astrocyte hypertrophy, proliferation, migration, acute mild inflammation) represent CNS physiological local response to limit the lesions, repair the initial damage, regulate homeostasis, prevent neurodegeneration and stimulate neurogenesis [45-47]. Indicative of astrocyte proliferation M phase Cell cycle function was increased in Romero-infected NHA at 24 h p.i. (Additional file 2: Table S2). On the other hand, massive/prolong brain injury or astrocyte stress lead to amplification of a microglia-astrocyte crosstalk and uncontrolled release of pro-inflammatory cytokines, chemokines and reactive oxygen species that has been associated with chronic neuroinflammation and neurodegeneration [45,46,48-50].

Multiple anti- and pro-inflammatory cytokines (i.e. IFN-α, IFN-β, IFN-γ, TNF-α, IL-1β, and IL-6), chemokines [i.e. stromal cell derived factor-1 alpha (SDF-1α, CXCL12), MCP-1, MIP-1α, RANTES and IP-10] and growth factors are produced by activated astroglia in response to an injury or pathogen [47]. Although, both neurogenesis and neurodegeneration has been associated with the factors produced by reactive astrocytes [49]; in our study transcriptom and cytokine/chemokine/growth factor profiles suggest the later response to JUNV infection. A link between chronic overproduction of type I IFNs and neurodegenerative diseases has been well established [50]. Likewise, we observed amplification with time of type I IFN signaling in response to JUNV infection. Moreover, in NHA we found elevated production/expression of pro-inflammatory mediators [type I cytokines (IL-12, INF-γ), proinflammatory cytokines (IL-6, TNF-α), chemoattractants and chemokines (IL-8, IL-10, MCP-1, RANTES)] but not anti-inflammatory cytokines (IL-4, IL-5, IL-10, IL-13, IL-17) upon JUNV infection. However, at 24 h p.i. we detected an elevated expression of SDF-1a, a key chemokine responsible for recruitment, proliferation and survival of neuronal precursor cells, and an elevated expression and production of FGF2 (BFGF) and VEGF at 24 and 96 h p.i. respectively (Figure 1, 3A) [49].

Supportive of exacerbated pro-inflammatory astrogliosis, Inflammatory and Neurological disease functions were uniformly increased at both time points in the microarray profile of JUNV-infected NHA. Among the others, neurological diseases such as relapsing-remitting multiple sclerosis and progressive motor neuropathy were listed. Pathogenic role of astrocytes in neurodegenerative disease including Amyotrophic lateral sclerosis has been documented [46,48,50].

The transcriptional profile of Romero JUNV-infected NHA indicated an apoptosis induction late in infection. Correspondingly, earlier we demonstrated IFN-independent RIG-I enhanced apoptosis induction in human and non-human primate transformed cells in response to virulent (Romero) and vaccine (Candid #1) strains of JUNV. Apoptosis induction in those cells was confirmed through quantification of phosphatidylserine translocation, Caspase 3 activation, Poly (ADP-ribose) polymerase (PARP) cleavage and/or chromosomal DNA fragmentation [51]. In agreement, activation of RIG-I/IRF3 pathway was evident in our microarray profile.

Importance of three branches of Death Receptor Signaling pathway CD95 (Fas)/CD95L (Fas L), TNF-TNFR1, and TNF-related apoptosis-inducing ligand (TRAIL) and it receptors has been described for cell death induction in the brain. Fas/FasL and TRAIL receptors are expressed by glia and neurons and responsible for triggering neuronal and astrocyte apoptosis; while Fas/FasL and TNF-TNFR1 pathways has been implicated in T-cell apoptosis induction [42,52,53]. In agreement with that, CASP6, CFLAR (FLIP), FADD, IKBKB, TNFSF10 (TRAIL), and TRADD transcripts were uniformly up-regulated at 96 h p.i. in Romero-infected NHA.

Complement components produced by glia interact with the apoptotic signals on the cell surface leading to complement receptor-dependent phagocytosis/clearance of apoptotic cells [42,54]. Accordingly, C3, C1R, C1S, CFH, CFI and SERPING1 encoding transcripts were up-regulated at 96 h p.i. in NHA infected with Romero JUNV. Detrimental inflammatory as well as neuroprotective role of complement activation has been reported [54].

The recent study [43] reported transcriptional changes of reactive astrocytes purified from mouse brains after ischemic stroke or LPS-induced neuroinflammation. Interestingly, the transcriptional profile of Romero-infected NHA in our study was similar to the detrimental pro-inflammatory molecular phenotype of reactive astrocytes induced by LPS, but not to the beneficial or protective response of reactive astrocytes in the ischemia model. Similar to our data, antigen presentation, complement, and IFN pathways were induced in the LPS reactive astrocytes. In contrast, increased metabolic activity, cell-cycle genes, and transcription factors categories were more prominent in reactive astrocytes from transient ischemia model [43].

In several previous studies of chronic encephalitis in newborn rats and mice induced by JUNV intracerebral inoculation or *in vitro* JUNV-infected rat and mouse astrocytes, a prominent astrocyte reaction to infection was reported [55-64]. In contrast to JUNV infection of human astrocytes, a protective role of JUNV-stimulated astrogliosis dependent on the increased expression of inducible nitric oxide synthase (iNOS) and not associated with the induction of apoptosis was reported in those studies [58,63]. This discrepancy could be attributed to the different mechanisms of JUNV interaction with cells of resistant or permissive hosts.

Although limited, autopsy findings of patients with AHF and pathology reports of AHF/LNS animal models suggest gliosis/astrogliosis and neuronal damage as part of the disease pathogenesis [17,19,20,26,27]. Our observation of neurodegenerative dysfunctional reactive astrogliosis in response to virulent strain of JUNV infection is in agreement with the human and animal model pathology. Additionally, production of proinflammatory cytokines (IL-6, TNF-α) and chemoattractant IL-8 by Romero-infected NHA reflects systemic cytokine profile of severely ill AHF patients [8,9]. Moreover, the transcriptional changes of multiple genes involved in IFN signaling detected in the JUNV-infected astrocytes partially corresponded to the *in vivo* transcriptional changes of blood mononuclear cells of Rhesus macaques infected with a virulent strain of LCMV [65], furthermore suggesting the relevance of *in vitr*o profile observed in our study to the *in vivo* response to JUNV.

In conclusion, our study clearly demonstrates for the first time the high susceptibility of human astrocytes to infection with hemorrhagic arenavirus. Additionally, our mRNA profile of JUNV-infected human astrocytes confirmed by qRT-PCR and cytokine/chemokine/growth factor assay suggests neurodegenerative dysfunctional reactive astrogliosis as a cellular response to this infection. Taken together, our results suggest potential role of astroglial cells in AHF pathogenesis, particularly pathogenesis of neurological symptoms and LNS.

## Competing interests

The authors declare that they have no competing interests.

## Authors’ contributions

OAK carried out the microarray, qRT-PCR and Bio-Plex assays, including data analysis, and drafted the manuscript. NEY carried out the infection and virus titration in the BSL4 and reviewed the manuscript. SP conceived of the study, and participated in its design and coordination and helped to draft the manuscript. All authors read and approved the final manuscript.

## Supplementary Material

Additional file 1: Table S1 Predicted activated and inhibited upstream transcriptional regulators in NHA infected with JUNV. Significance of Upstream Regulator Analysis was based on combination of the activation z-score (-2 ≥ Z ≥ 2) and overlap P-value (P < 0.05). Predicted activation state is depicted in green for inhibited or in red for activated upstream transcriptional regulators. Upstream transcriptional regulators that are likely involved into regulation of gene expression at 24 h p.i. only are not highlighted, at 96 h p.i. only are highlighted in light grey, at 24 and 96 h p.i. – highlighted in dark grey. Click here for file

Additional file 2: Table S2 Predicted increase or decrease of biological functions in NHA in response to JUNV infection at 24 h p.i. Significance of Downstream Effects analysis was based on combination of the activation z-score (-2 ≥ Z ≥ 2) and overlap P-value (P < 0.05). Click here for file

Additional file 3: Table S3 Predicted increase or decrease of biological functions in NHA in response to JUNV infection at 96 h p.i. Significance of Downstream Effects analysis was based on combination of the activation z-score (-2 ≥ Z ≥ 2) and overlap P-value (P < 0.05). Cell death functions are highlighted in grey. Click here for file

Additional file 4: Table S4 High correlation of microarray and qRT-PCR data for a subset of immune response genes. Confirmation of expression of selected differentially expressed (array) IFN signaling genes at 24 and 96 h post JUNV infection of NHA by qRT-PCR using cDNA from JUNV-infected NHA and from mock-infected control cells. P-values originated from statistical analysis of fold-changes for qRT-PCR are provided in the table. ND, not determined. Click here for file
