# Supplementary material for: Reactive astrogliosis in response to hemorrhagic fever virus: microarray profile of Junin virus-infected human astrocytes
Source: Virol J. 2014 Jul 11;11:126. doi: 10.1186/1743-422X-11-126 (PMC4113780; doi:10.1186/1743-422X-11-126)
Supplement: Additional file 1: Table S1 — Predicted activated and inhibited upstream transcriptional regulators in NHA infected with JUNV. Significance of Upstream Regulator Analysis was based on combination of the activation z-score (-2 ≥ Z ≥ 2) and overlap P-value (P < 0.05). Predicted activation state is depicted in green for inhibited or in red for activated upstream transcriptional regulators. Upstream transcriptional regulators that are likely involved into regulation of gene expression at 24 h p.i. only are not highlighted, at 96 h p.i. only are highlighted in light grey, at 24 and 96 h p.i. – highlighted in dark grey. [file 1743-422X-11-126-S1.docx]

| **Upstream regulator** | **Hours p.i.** | **Log ratio** | **Predicted activation state** | **Activation z-score** | **P-value of overlap** | **Target molecules in dataset** |
| --- | --- | --- | --- | --- | --- | --- |
| BRCA1 | 24 |  | Activated | 2.600 | 9.02E-13 | DDX58,H3F3A/H3F3B,IFI27,IFI6,IFIT1,IFIT3,IFITM1,IRF7,MX1,NEK2,PERP,PLSCR1 |
| bromodeoxyuridine | 24 |  | Activated | 2.789 | 1.17E-10 | IFI27,IFI6,IFIT1,IFIT3,IFITM1,IRF7,MX1,OAS1 |
| decitabine | 24 |  | Activated | 2.269 | 8.84E-04 | BTG1,COL1A1,IFI27,IFITM1,IGFBP7,IRF7,MX1,OAS1 |
| E. coli B4 lipopolysaccharide | 24 |  | Activated | 2.164 | 6.57E-03 | DDX58,IFIT3,MDFIC,RSAD2,USP18 |
| imiquimod | 24 |  | Activated | 2.446 | 5.45E-07 | IFITM1,IRF7,MX1,OAS1,OASL,RSAD2 |
| Jnk | 24 |  | Activated | 2.177 | 1.05E-03 | APP,BTG1,COL1A1,CXCL12,PLSCR1 |
| lipopolysaccharide | 24 |  | Activated | 3.829 | 1.85E-10 | APOBEC3G,APP,ATP2A2,BTG1,COL1A1,DDX58,ELK3,FGF2,IFI27,IFI44L,IFI6,IFIT1,IFIT3,IFITM1,IRF7,MARCKS,MX1,MX2,OAS1,OAS2,OAS3,OASL,PERP,PLSCR1,PPP1CB,RARRES3,RSAD2,USP18 |
| MYD88 | 24 |  | Activated | 2.085 | 3.44E-03 | CXCL12,IRF7,OASL,RSAD2,USP18 |
| NFATC2 | 24 |  | Activated | 2.000 | 4.02E-03 | IFIT3,IRF7,OASL,RSAD2 |
| NFkB (complex) | 24 |  | Activated | 2.000 | 4.08E-02 | APP,ATP2A2,CXCL12,FGF2,IRF7,RSAD2 |
| PAF1 | 24 |  | Activated | 2.449 | 6.04E-08 | DDX58,IFI44L,IFIT3,OAS2,OAS3,OASL |
| phorbol myristate acetate | 24 |  | Activated | 2.714 | 9.83E-03 | APOBEC3G,APP,ATP2A2,CPE,FGF2,IRF7,PPP1R12A,PRKAR1A,PRNP,RBBP4 |
| poly rI:rC-RNA | 24 |  | Activated | 3.484 | 2.18E-08 | APOBEC3G,DDX58,FGF2,IFI27,IFIT1,IFIT3,IRF7,MX1,OAS1,OAS2,OAS3,RARRES3,RSAD2 |
| ribavirin | 24 |  | Activated | 3.000 | 1.42E-14 | DDX58,IFI27,IFI44L,IFIT3,IFITM1,IRF7,MX1,OASL,USP18 |
| stallimycin | 24 |  | Activated | 2.789 | 3.02E-11 | IFI27,IFI6,IFIT1,IFIT3,IFITM1,IRF7,MX1,OAS1 |
| TGM2 | 24 |  | Activated | 2.714 | 5.05E-09 | DDX60,IFI6,IFIT1,IFIT3,OAS1,OAS2,OAS3,OASL,PLSCR1,RARRES3,SACS |
| TNF | 24 |  | Activated | 2.958 | 8.13E-09 | APP,ATP2A2,BTG1,BUB1B,CELF1,COL1A1,CXCL12,DDX58,ELK3,FGF2,IFI27,IFI6,IFIT1,IFIT3,IFITM1,IRF7,KIF20A,MX1,OAS1,OAS2,OAS3,OASL,PLSCR1,PRNP,RARRES3 |
| tretinoin | 24 |  | Activated | 2.668 | 3.99E-09 | APP,ATP2A2,BTG1,COL1A1,CPE,DDX58,DDX60,IFI27,IFI6,IFIT1,IFIT3,IFITM1,IGFBP7,MARCKS,OAS1,OAS2,OAS3,OASL,PLS3,PLSCR1,PRNP,RARRES3,SACS,USP18 |
| ADRB | 24 |  | Inhibited | -2.000 | 3.27E-03 | IFIT3,NEK2,OASL,RSAD2 |
| CLDN7 | 24 |  | Inhibited | -2.000 | 2.26E-03 | IFI6,IGFBP7,MX1,PRNP |
| GAPDH | 24 |  | Inhibited | -2.236 | 3.17E-07 | IFI6,IFITM1,OAS1,OAS2,OAS3 |
| bisphenol A | 96 |  | Activated | 2.200 | 1.68E-02 | C3,CTGF,CTTN,EGR1,IKBKB |
| bromodeoxyuridine | 96 |  | Activated | 2.985 | 2.29E-07 | IFI27,IFI44,IFI6,IFIT1,IFITM1,ISG15,MX1,OAS1,TNFSF10 |
| CHUK | 96 |  | Activated | 2.397 | 1.82E-03 | C3,DUSP6,IFI35,IGFBP6,ISG15,PCDH7,PLSCR1,SQSTM1,TNFRSF11B,VEGFA |
| decitabine | 96 |  | Activated | 2.266 | 1.31E-06 | BTG1,CDH11,CDH6,CDKN1C,CHI3L1,COPA,CTGF,DIRAS3,DLC1,DUSP1,IFI27,IFI35,IFITM1,ISG15,MED7,MX1,NPTX1,NQO1,OAS1,PRDM2,RRAD,RTP4,TGFBR2 |
| IFNgamma | 96 |  | Activated | 2.201 | 1.28E-02 | CASP1,IFI44L,MYD88,PCDH7,TNFSF10 |
| IKBKB | 96 | 3.894 | Activated | 2.407 | 7.12E-04 | C3,DUSP6,EGR1,FYN,GRK5,IGFBP6,IKBKB,ISG15,MX1,PCDH7,TNFRSF11B,VEGFA |
| IKBKG | 96 |  | Activated | 2.202 | 1.07E-03 | C3,CFLAR,DUSP6,IGFBP6,ISG15,PCDH7,TNFSF10,VEGFA |
| imiquimod | 96 |  | Activated | 2.993 | 1.34E-06 | ICAM3,IFI35,IFITM1,ISG20,MX1,OAS1,OASL,RSAD2,TNFSF10 |
| JAK1 | 96 |  | Activated | 2.000 | 3.15E-03 | ID1,IRF9,MX1,USP18 |
| lenalidomide | 96 |  | Activated | 2.000 | 1.63E-01 | AUTS2,CASP1,COX11,RTP4 |
| lipopolysaccharide | 96 |  | Activated | 2.545 | 1.91E-12 | ADM,ARG2,ARL4C,BTG1,C3,CASP1,CD55,CDC42EP2,CDH11,CFLAR,CHI3L1,CLIC3,CRYAB,CTGF,DDIT3,DDX58,DLG4,DUSP1,EGR1,EGR2,FYN,GSPT2,ID2,IFI27,IFI35,IFI44,IFI44L,IFI6,IFIT1,IFITM1,INSIG1,IRF9,ISG15,ISG20,KCNK1,LITAF,MICA,MX1,MX2,MYD88,NGFR,NPTX1,NQO1,NRP1,OAS1,OAS2,OAS3,OASL,PANX1,PCDH7,PFKFB3,PIM1,PLSCR1,PPP1R15A,PSMA2,RARRES3,RSAD2,TFDP1,TNFSF10,TNFSF18,TRADD,TRAF3,TUBB4B,USP18,VEGFA,ZFP36 |
| NR3C2 | 96 |  | Activated | 2.236 | 1.61E-02 | CTGF,EGR1,RRAD,TSPYL4,VEGFA |
| OSM | 96 |  | Activated | 2.459 | 4.47E-05 | ARL4C,C1R,C1S,GLUL,ID1,ID2,IFI35,IGFBP6,IRF9,ISG20,LITAF,MICA,MKNK2,MLLT11,MX1,MYD88,NUAK1,OAS1,PFKFB3,SERPING1,TNFRSF11B,VEGFA |
| PAF1 | 96 |  | Activated | 2.333 | 5.33E-08 | DDX58,IFI44,IFI44L,ISG15,ISG20,OAS2,OAS3,OASL,ZFP36 |
| PARP9 | 96 |  | Activated | 2.000 | 1.14E-04 | IFI44,IFIT1,ISG15,OAS2 |
| poly rI:rC-RNA | 96 |  | Activated | 3.824 | 3.66E-10 | BST2,C3,CFLAR,DDX58,DUSP1,EGR1,EZR,IFI27,IFIT1,IGFBP6,IRF9,ISG15,ISG20,MX1,MYD88,OAS1,OAS2,OAS3,PIM1,PPP1R15A,RARRES3,RNF114,RSAD2,SERPING1,TNFRSF11B,TNFSF10,ZFP36 |
| PTEN | 96 |  | Activated | 2.378 | 1.60E-06 | ADM,BTG1,C3,DUSP1,EGR2,GCAT,GTF2I,HDAC5,IGFBP6,ISG15,MAT2A,MDM2,NGFR,PABPC4,PIM1,PTP4A2,SCD,TNFRSF11B,TNFSF10,TRADD,VEGFA |
| ribavirin | 96 |  | Activated | 2.813 | 3.82E-08 | DDX58,IFI27,IFI44L,IFITM1,ISG15,MX1,OASL,USP18 |
| Salmonella enterica serotype abortus equi lipopolysaccharide | 96 |  | Activated | 2.646 | 2.32E-02 | CFLAR,EGR1,FJX1,ID2,ISG15,OASL,RIN2 |
| salmonella minnesota R595 lipopolysaccharides | 96 |  | Activated | 2.214 | 1.99E-02 | CFLAR,EGR1,ISG15,OASL,RSAD2 |
| SMAD4 | 96 |  | Activated | 2.130 | 7.19E-04 | CTGF,EGFR,ID1,ID2,MDM2,RGCC,SCD,SLC25A4,VEGFA,ZFP36 |
| stallimycin | 96 |  | Activated | 2.985 | 5.33E-08 | IFI27,IFI44,IFI6,IFIT1,IFITM1,ISG15,MX1,OAS1,TNFSF10 |
| TLR4 | 96 |  | Activated | 2.190 | 2.12E-02 | ADM,C3,ISG15,ISG20,MX1,MYD88,OASL,RGCC,RSAD2,TNFSF10 |
| TNFSF10 | 96 | 3.548 | Activated | 2.781 | 7.00E-07 | CFLAR,EGFR,FADD,IFI6,IFIT1,IFITM1,IRF9,ISG15,TNFSF10,VEGFA |
| tretinoin | 96 |  | Activated | 2.859 | 2.58E-09 | ADM,BTG1,C3,CASP1,CASP6,CDH6,COX11,CTGF,DDIT3,DDX58,DDX60,DLC1,DNAAF2,DUSP1,EGFR,EGR1,ELF4,GLI2,GSPT2,ICAM3,ID1,ID2,IFI27,IFI35,IFI6,IFIT1,IFITM1,IGFBP6,IRF9,ISG15,MDM2,NRP1,OAS1,OAS2,OAS3,OASL,PIM1,PLSCR1,PRKCDBP,PTGIS,RARRES3,RUVBL1,SACS,SCD,SERPING1,SNX19,SOX9,SPEN,TGFBR2,TNFRSF11B,TNFSF10,USP18,VEGFA |
| 2-aminopurine | 96 |  | Inhibited | -2.225 | 3.16E-05 | DDX58,IFI6,IFIT1,ISG15,OAS1 |
| CD3 | 96 |  | Inhibited | -2.004 | 2.06E-05 | ANG,BST2,BTG1,C1R,CASP6,CFLAR,EGR2,FYN,GTF2IRD1,HNRNPA1,IFI35,IFIT1,IFITM1,IGFBP6,IRF9,MICA,PABPC4,PTP4A2,RRAGA,SRSF1,TARDBP,TNFSF10,TNFSF18,TPST1,TRIP10,ZFP36 |
| dihydrotestosterone | 96 |  | Inhibited | -2.143 | 4.12E-04 | ADM,CASP1,CHI3L1,CSNK2A1,CTGF,DLG4,EGFR,EGR1,GLUL,GOSR1,GTF2I,ID2,LDHA,MDM2,PER1,SCD,SRGAP2,TGFBR2,VEGFA |
| epigallocatechin-gallate | 96 |  | Inhibited | -2.154 | 7.19E-04 | CASP1,CASP6,CFLAR,CTGF,EGFR,EGR1,FADD,ISG15,MDM2,TNFSF10,VEGFA |
| fontolizumab | 96 |  | Inhibited | -2.000 | 1.47E-04 | IFI35,ISG15,MX1,RSAD2 |
| GFI1 | 96 |  | Inhibited | -2.369 | 2.86E-03 | CASP1,CFLAR,ID2,IKBKB,TRADD,TRAF3 |
| KDM5B | 96 |  | Inhibited | -2.867 | 4.60E-07 | ASF1A,C20orf111,DDIT3,EGR1,FJX1,INSIG1,ISG15,NEDD9,PHF15,SOX9,SPTSSA,TUBB2A |
| Lh | 96 |  | Inhibited | -2.142 | 1.32E-05 | ARL4C,CD55,CDK14,CDKN1C,CFLAR,DUSP1,EGFR,EZR,GPR56,GRK5,PER1,PTPRE,TNFRSF11B,UPP1,VEGFA |
| methylprednisolone | 96 |  | Inhibited | -2.029 | 3.16E-02 | ABAT,BTG1,CSNK2A1,CTGF,DUSP1,DUSP6,GCLM,ID1,IDH2,INSIG1,RGCC,RUVBL1,SERPINB7,UBE2G2,ZFP36 |
| NUPR1 | 96 |  | Inhibited | -5.209 | 2.64E-10 | ADM,BTG1,C20orf111,CSTF2T,DDIT3,FAM114A1,GPR56,GSTA4,HNRNPM,MAT2A,MKL2,MX2,MYD88,NAA40,NGFR,PFKFB3,PHF15,PIM1,PPP1R15A,RFX5,SIK1,SPATS2L,SPG7,SPTSSA,SRGAP2,SRSF1,STK38,TARDBP,UBIAD1,UPP1,VANGL1 |
| prednisolone | 96 |  | Inhibited | -2.137 | 1.46E-03 | BTG1,CFLAR,DUSP1,DUSP10,FADD,ISG20,RARRES3,SIK1,SQSTM1,TNFRSF11B,TNFSF10 |
| SOCS3 | 96 |  | Inhibited | -2.425 | 1.05E-03 | EGR1,IFIT1,ISG20,MX1,OAS1,OAS2 |
| TP63 | 96 |  | Inhibited | -2.132 | 8.67E-02 | ADM,BST2,DUSP10,ID1,IGFBP6,MDM2,VEGFA |
| USP18 | 96 | 4.103 | Inhibited | -2.393 | 2.07E-07 | IFI6,IRF9,ISG15,MX1,OAS1,TNFSF10 |
| EIF2AK2 | 24 |  | Activated | 2.985 | 1.10E-10 | DDX58,IFI27,IFI6,IFIT1,IFITM1,OAS1,OAS3,PLSCR1,USP18 |
|  | 96 |  | Activated | 2.882 | 1.69E-14 | DDIT3,DDX58,EGR1,IFI27,IFI35,IFI6,IFIT1,IFITM1,ISG15,ISG20,NEDD9,OAS1,OAS3,PARP12,PLSCR1,PPP1R15A,USP18 |
| IFN | 24 |  | Activated | 2.934 | 3.78E-11 | DDX58,IFIT1,IFITM1,IRF7,MX1,OAS2,OAS3,OASL,RARRES3,RSAD2 |
|  | 96 |  | Activated | 3.253 | 1.36E-07 | DDX58,IFIT1,IFITM1,ISG15,ISG20,MICA,MX1,OAS2,OAS3,OASL,RARRES3,RSAD2 |
| IFN beta | 24 |  | Activated | 2.782 | 4.02E-08 | IFI6,IFIT1,IRF7,MX1,OAS1,OAS2,RSAD2,USP18 |
|  | 96 |  | Activated | 3.096 | 2.32E-06 | IFI6,IFIT1,INSIG1,IRF9,ISG15,MX1,OAS1,OAS2,RSAD2,TNFSF10,USP18 |
| IFNA1/IFNA13 | 24 |  | Activated | 3.369 | 5.33E-17 | IFI27,IFI6,IFIT1,IFITM1,MX1,OAS1,OAS2,OASL,PLSCR1,RARRES3,RSAD2,USP18 |
|  | 96 |  | Activated | 3.498 | 1.76E-11 | IFI27,IFI6,IFIT1,IFITM1,ISG15,MX1,OAS1,OAS2,OASL,PLSCR1,RARRES3,RSAD2,USP18 |
| IFNA2 | 24 |  | Activated | 3.804 | 2.94E-19 | DDX58,DDX60,FGF2,IFI27,IFI44L,IFI6,IFIT1,IFIT3,IFITM1,IRF7,MX1,MX2,OAS1,OAS2,OAS3,PLSCR1,RARRES3,RSAD2,USP18 |
|  | 96 |  | Activated | 5.058 | 6.29E-20 | BST2,C1R,C1S,DDX58,DDX60,HERC6,IFI27,IFI35,IFI44,IFI44L,IFI6,IFIT1,IFITM1,IRF9,ISG15,ISG20,MDM2,MX1,MX2,OAS1,OAS2,OAS3,PARP12,PARP4,PLSCR1,RARRES3,RSAD2,TNFSF10,TRIM14,USP18,VEGFA |
| IFNAR1 | 24 |  | Activated | 2.360 | 1.76E-08 | IFIT3,IRF7,OAS1,OAS2,OAS3,OASL,RSAD2,USP18 |
|  | 96 |  | Activated | 2.164 | 4.21E-05 | IFI44,ISG15,OAS1,OAS2,OAS3,OASL,RSAD2,RTP4,USP18 |
| IFNB1 | 24 |  | Activated | 2.269 | 1.52E-09 | DDX58,FGF2,IFI6,IFIT1,IFIT3,IFITM1,IRF7,MX1,OAS1,RARRES3,RSAD2,USP18 |
|  | 96 |  | Activated | 3.524 | 1.05E-06 | BST2,CASP1,CRYAB,DDX58,IFI6,IFIT1,IFITM1,IRF9,ISG15,MX1,MYD88,NPTX1,OAS1,RARRES3,RSAD2,TNFSF10,USP18 |
| IFNG | 24 |  | Activated | 4.003 | 4.94E-11 | APOBEC3G,APP,ATP2A2,BTG1,COL1A1,CXCL12,DDX58,FGF2,IFI27,IFI44L,IFI6,IFIT1,IFIT3,IFITM1,IRF7,MX1,MX2,OAS1,OAS2,OAS3,OASL,PRNP,RARRES3,RSAD2,USP18 |
|  | 96 |  | Activated | 4.589 | 4.44E-18 | ADM,ARG2,AUTS2,BST2,BTG1,C1R,C3,CASP1,CASP6,CD55,CFLAR,CLIP2,CTGF,DDIT3,DDX58,DUSP1,EGR1,EGR2,F11R,GLUL,GPR56,HCP5,HERC6,ID1,IFI27,IFI35,IFI44,IFI44L,IFI6,IFIT1,IFITM1,IRF9,ISG15,ISG20,LDHA,MAT2A,MICA,MX1,MX2,MYD88,NEDD9,NQO1,OAS1,OAS2,OAS3,OASL,PANX1,PIM1,PSMA2,RARRES3,RFX5,RRAGD,RSAD2,RTP4,SERPING1,SQSTM1,TAPBPL,TFDP1,TGFBR2,TNFRSF11B,TNFSF10,TTC28,USP18,VEGFA,ZFP36 |
| IFNL1 | 24 |  | Activated | 4.051 | 5.81E-25 | DDX58,DDX60,IFI27,IFI44L,IFI6,IFIT1,IFIT3,IFITM1,MX1,OAS1,OAS2,OAS3,OASL,PLSCR1,RARRES3,RSAD2,USP18 |
|  | 96 |  | Activated | 4.831 | 6.27E-25 | BST2,DDX58,DDX60,HERC6,IFI27,IFI35,IFI44,IFI44L,IFI6,IFIT1,IFITM1,IRF9,ISG15,ISG20,MX1,OAS1,OAS2,OAS3,OASL,PLSCR1,RARRES3,RSAD2,TRIM14,USP18 |
| IFN alpha | 24 |  | Activated | 3.817 | 1.57E-11 | APOBEC3G,DDX58,IFI27,IFI6,IFIT1,IFIT3,IFITM1,IRF7,MX1,MX2,OAS1,OAS2,RARRES3,RSAD2,USP18 |
|  | 96 |  | Activated | 4.400 | 2.86E-11 | AUTS2,BST2,C3,CASP1,DDX58,EGFR,IFI27,IFI35,IFI6,IFIT1,IFITM1,IRF9,ISG15,ISG20,MDM2,MX1,MX2,MYD88,OAS1,OAS2,PIM1,RARRES3,RSAD2,TNFSF10,TTC28,USP18 |
| IRF1 | 24 |  | Activated | 2.779 | 1.38E-09 | IFI44L,IFIT1,IFIT3,IFITM1,IRF7,MX1,OAS1,OAS2,RARRES3,RSAD2 |
|  | 96 |  | Activated | 3.393 | 2.49E-08 | C1R,CASP1,IFI35,IFI44L,IFIT1,IFITM1,IRF9,ISG15,MX1,OAS1,OAS2,PCDH7,RARRES3,RSAD2,TNFSF10 |
| IRF3 | 24 |  | Activated | 2.773 | 4.02E-13 | DDX58,IFI6,IFIT1,IFIT3,IRF7,MARCH6,OAS1,OAS2,OAS3,OASL,PRNP,RSAD2,USP18 |
|  | 96 |  | Activated | 3.423 | 1.53E-09 | ANXA4,ARG2,CDH11,DDX58,IFI44,IFI6,IFIT1,ISG15,ISG20,OAS1,OAS2,OAS3,OASL,PARP12,RSAD2,TPST1,USP18 |
| IRF5 | 24 |  | Activated | 2.928 | 2.19E-11 | DDX58,IFIT1,IFIT3,IRF7,OAS1,OAS2,OASL,PLSCR1,RSAD2 |
|  | 96 |  | Activated | 3.382 | 1.09E-09 | DDX58,IFI44,IFIT1,ISG15,ISG20,OAS1,OAS2,OASL,PARP12,PLSCR1,RSAD2,TNFSF10 |
| IRF7 | 24 | 3.065 | Activated | 4.024 | 5.77E-19 | APOBEC3G,DDX58,IFI44L,IFI6,IFIT1,IFIT3,IFITM1,IRF7,MX1,MX2,OAS1,OAS2,OAS3,OASL,PLSCR1,RSAD2,USP18 |
|  | 96 |  | Activated | 4.675 | 2.01E-15 | DDX58,IFI35,IFI44,IFI44L,IFI6,IFIT1,IFITM1,IRF9,ISG15,ISG20,MX1,MX2,OAS1,OAS2,OAS3,OASL,PARP12,PLSCR1,RSAD2,RTP4,TNFSF10,TPST1,USP18 |
| MAVS | 24 |  | Activated | 2.805 | 1.17E-10 | DDX58,IFIT1,IFIT3,IRF7,OAS1,OAS2,OASL,RSAD2 |
|  | 96 |  | Activated | 2.973 | 2.29E-07 | DDX58,IFIT1,ISG15,ISG20,OAS1,OAS2,OASL,PARP12,RSAD2 |
| SAMSN1, SLy2 | 24 |  | Activated | 2.000 | 7.90E-04 | IFIT3,IRF7,OASL,RSAD2 |
|  | 96 |  | Activated | 2.236 | 1.76E-02 | ISG15,ISG20,OASL,RGCC,RSAD2 |
| SASH1 | 24 |  | Activated | 2.000 | 3.08E-04 | IFIT3,IRF7,OASL,RSAD2 |
|  | 96 |  | Activated | 2.000 | 3.00E-02 | ISG15,ISG20,OASL,RSAD2 |
| STAT1 | 24 |  | Activated | 2.261 | 1.21E-09 | APOBEC3G,BTG1,FGF2,IFI27,IFI6,IFIT1,IFIT3,IFITM1,IRF7,OASL,RSAD2,USP18 |
|  | 96 |  | Activated | 3.205 | 9.34E-10 | BTG1,C3,CASP1,CASP6,EGR1,HERC6,IFI27,IFI35,IFI6,IFIT1,IFITM1,IRF9,ISG15,MDM2,OASL,PIM1,RSAD2,SERPING1,TAPBPL,TNFSF10,USP18 |
| STAT2 | 24 |  | Activated | 2.393 | 1.42E-14 | IFI27,IFI6,IFIT1,IFIT3,IFITM1,IRF7,MX1,OAS1,RSAD2 |
|  | 96 |  | Activated | 2.764 | 2.03E-12 | IFI27,IFI35,IFI6,IFIT1,IFITM1,IRF9,ISG15,MX1,OAS1,RSAD2,TNFSF10 |
| TGFB1 | 24 |  | Activated | 2.401 | 2.47E-04 | APP,BTG1,BUB1B,CDK17,COL1A1,CXCL12,ELK3,FGF2,IFI27,IFIT3,IGFBP7,NEK2,PGK1,PLS3,PLSCR1,RAD21,RSAD2,TPM1 |
|  | 96 |  | Activated | 2.647 | 4.20E-10 | ADM,ARID5B,BTG1,C1S,CALML4,CASP1,CD55,CDH11,CDKN1C,CHI3L1,COL16A1,CTGF,CTPS1,CTTN,DNAJB6,DUSP1,EGR1,EGR2,ELF4,FILIP1L,FUS,FYN,GLI2,ID1,ID2,IFI27,IGFBP6,LDHA,LITAF,MDM2,MYD88,NEDD9,NPAS2,NRP1,NUAK1,PHF15,PIM1,PLSCR1,RGCC,RRAD,RSAD2,SAR1A,SCD,SLC25A4,SOX9,SPRY1,SRR,SRSF2,SRSF5,ST3GAL5,TARDBP,TENM4,TGFBR2,TLE4,TNFRSF11B,TTC28,TUBB2A,TUBB4B,VEGFA,XRCC4,ZFP36 |
| TICAM1, TRIF | 24 |  | Activated | 2.415 | 3.51E-05 | DDX58,IFIT1,IFIT3,IRF7,OASL,RSAD2 |
|  | 96 |  | Activated | 2.959 | 8.46E-05 | CFLAR,DDX58,DUSP1,EGR1,IFIT1,ISG15,ISG20,OASL,RSAD2,TNFSF10 |
| TLR3 | 24 |  | Activated | 2.621 | 3.76E-11 | DDX58,IFI44L,IFI6,IFIT1,IFIT3,IRF7,MARCKS,MX1,MX2,OAS1,OASL,RSAD2,USP18 |
|  | 96 |  | Activated | 2.226 | 4.06E-11 | ARG2,C3,DDX58,DUSP1,ID1,IFI44,IFI44L,IFI6,IFIT1,ISG15,ISG20,MX1,MX2,MYD88,OAS1,OASL,PFKFB3,RSAD2,SPRY1,TNFSF10,TRAF3,USP18 |
| IL1RN | 24 |  | Inhibited | -3.742 | 1.79E-16 | DDX58,IFI27,IFI44L,IFI6,IFIT3,IRF7,MX1,MX2,OAS1,OAS2,OAS3,OASL,RSAD2,USP18 |
|  | 96 |  | Inhibited | -4.243 | 8.13E-13 | DDX58,HERC6,IFI27,IFI44,IFI44L,IFI6,IRF9,ISG20,MX1,MX2,OAS1,OAS2,OAS3,OASL,RSAD2,RTP4,TNFSF10,USP18 |
| IRGM | 24 |  | Inhibited | -2.828 | 1.70E-11 | IFIT3,IRF7,KIF20A,NEK2,OAS2,OASL,RSAD2,USP18 |
|  | 96 |  | Inhibited | -2.236 | 6.89E-04 | ID2,OAS2,OASL,RSAD2,USP18 |
| KRAS | 24 |  | Inhibited | -2.621 | 5.22E-04 | COL1A1,IFI6,IFIT1,IFITM1,MX1,MX2,OAS1 |
|  | 96 |  | Inhibited | -2.883 | 1.08E-08 | CASP1,CRYAB,DDIT3,DUSP6,GTF2I,ID1,IFI6,IFIT1,IFITM1,IRF9,ISG15,MDM2,MX1,MX2,NQO1,OAS1,PIM1,SQSTM1,TGFBR2,TNFRSF11B,UPP1,VEGFA |
| let-7 | 24 |  | Inhibited | -2.000 | 2.07E-03 | BUB1B,COL1A1,DZIP1,MCM4 |
|  | 96 |  | Inhibited | -2.414 | 1.37E-02 | DLC1,GTF2I,ID2,MYD88,SOX9,ZNF512B |
| MAPK1, ERK2 | 24 |  | Inhibited | -3.988 | 9.11E-13 | DDX58,IFI27,IFI6,IFIT1,IFIT3,IFITM1,IGFBP7,IRF7,MX2,OAS1,OAS2,OAS3,OASL,PAIP1,PLSCR1,USP18 |
|  | 96 |  | Inhibited | -4.427 | 3.37E-12 | BST2,C1S,DDX58,DUSP1,EGR1,EGR2,IFI27,IFI35,IFI44,IFI6,IFIT1,IFITM1,IRF9,ISG15,ISG20,MX2,NRP1,OAS1,OAS2,OAS3,OASL,PARP12,PLSCR1,TNFSF10,TRIM14,TRIM34,USP18 |
| SB203580 | 24 |  | Inhibited | -2.803 | 1.24E-04 | APOBEC3G,COL1A1,IFI27,IFIT1,IFIT3,IRF7,MX1,RARRES3 |
|  | 96 |  | Inhibited | -2.293 | 6.22E-07 | BST2,C3,CTGF,DDIT3,DUSP1,EGR1,GSTA4,IFI27,IFI35,IFIT1,ISG15,ISG20,MX1,NQO1,PPP1R15A,RARRES3,SERPING1,TNFRSF11B,TNFSF10,VEGFA |
| SOCS1 | 24 |  | Inhibited | -2.391 | 7.34E-07 | DDX58,IFIT1,IFIT3,IRF7,MX1,OAS1,OAS2 |
|  | 96 |  | Inhibited | -2.374 | 9.54E-05 | DDX58,DUSP1,IFI44,IFIT1,ISG15,ISG20,MX1,OAS1,OAS2 |
| TRIM24 | 24 |  | Inhibited | -2.621 | 2.92E-07 | DDX58,DDX60,IFIT3,IRF7,OAS1,OASL,USP18 |
|  | 96 |  | Inhibited | -3.850 | 5.14E-12 | BST2,CFHR1,DDX58,DDX60,GLUL,HERC6,IFI35,IFI44,IRF9,ISG15,OAS1,OASL,PARP12,RTP4,USP18,VEGFA |
