# Supplementary material for: Reactive astrogliosis in response to hemorrhagic fever virus: microarray profile of Junin virus-infected human astrocytes
Source: Virol J. 2014 Jul 11;11:126. doi: 10.1186/1743-422X-11-126 (PMC4113780; doi:10.1186/1743-422X-11-126)
Supplement: Additional file 2: Table S2 — Predicted increase or decrease of biological functions in NHA in response to JUNV infection at 24 h p.i. Significance of Downstream Effects analysis was based on combination of the activation z-score (-2 ≥ Z ≥ 2) and overlap P-value (P < 0.05). [file 1743-422X-11-126-S2.docx]

| **Category** | **Functions annotation** | **p-Value** | **Predicted activation state** | **Activation z-score** | **Molecules** | **Number of molecules** |
| --- | --- | --- | --- | --- | --- | --- |
| Cancer | infection of tumor cell lines | 5.21E-03 | Increased | 2.145 | ARCN1,CTBP1,DDX58,H3F3A/H3F3B,IFITM1,LRIF1,POLR2K,PRNP,SYNJ2 | 9 |
| Cell Cycle | M phase | 1.04E-03 | Increased | 2.000 | APP,BUB1B,KIF20A,MCM4,NEK2,TPM1 | 6 |
| Cell Signaling | concentration of Ca2+ | 4.04E-04 | Increased | 2.200 | APP,ATP2A2,CXCL12,FGF2,RAB4A | 5 |
| Cell Signaling | replication of viral replicon | 7.42E-07 | Decreased | -2.213 | APOBEC3G,MX1,OAS1,OAS3,PLSCR1 | 5 |
| Infectious Disease | infection of tumor cell lines | 5.21E-03 | Increased | 2.145 | ARCN1,CTBP1,DDX58,H3F3A/H3F3B,IFITM1,LRIF1,POLR2K,PRNP,SYNJ2 | 9 |
| Infectious Disease | replication of Herpesviridae | 3.51E-04 | Decreased | -2.000 | DDX58,MX2,OAS1,RSAD2 | 4 |
| Infectious Disease | replication of vesicular stomatitis virus | 7.01E-07 | Decreased | -2.111 | APOBEC3G,DDX58,OAS1,OASL,PPM1B,RARRES3 | 6 |
| Infectious Disease | replication of Hepatitis C virus | 9.31E-07 | Decreased | -2.384 | DDX58,IFI27,IFI6,IFIT1,IFITM1,RSAD2 | 6 |
| Infectious Disease | replication of RNA virus | 7.33E-09 | Decreased | -2.743 | APOBEC3G,ARCN1,CTBP1,CXCL12,DDX58,IFI27,IFI6,IFIT1,IFITM1,IRF7,MX1,OAS1,OASL,PPM1B,PRNP,RARRES3,RSAD2 | 17 |
| Infectious Disease | replication of virus | 4.49E-09 | Decreased | -2.894 | APOBEC3G,ARCN1,CTBP1,CXCL12,DDX58,IFI27,IFI6,IFIT1,IFITM1,IRF7,MX1,MX2,OAS1,OASL,PPM1B,PRNP,RARRES3,RSAD2 | 18 |
| Inflammatory Disease | relapsing-remitting multiple sclerosis | 1.42E-08 | Increased | 2.433 | IFIT1,IRF7,MX1,OAS1,OAS3,RSAD2 | 6 |
| Inflammatory Disease | multiple sclerosis | 4.14E-05 | Increased | 2.433 | APP,IFIT1,IRF7,MX1,OAS1,OAS3,RSAD2 | 7 |
| Molecular Transport | concentration of Ca2+ | 4.04E-04 | Increased | 2.200 | APP,ATP2A2,CXCL12,FGF2,RAB4A | 5 |
| Neurological Disease | relapsing-remitting multiple sclerosis | 1.42E-08 | Increased | 2.433 | IFIT1,IRF7,MX1,OAS1,OAS3,RSAD2 | 6 |
| Neurological Disease | neuromuscular disease | 5.95E-06 | Increased | 2.433 | APP,ATP2A2,FGF2,H3F3A/H3F3B,IFIT1,IRF7,MRPL9,MX1,OAS1,OAS3,PGK1,PPM1B,PPP1CB,PRNP,RARRES3,RSAD2,SACS,SEP15 | 18 |
| Neurological Disease | progressive motor neuropathy | 3.40E-05 | Increased | 2.433 | APP,H3F3A/H3F3B,IFIT1,IRF7,MX1,OAS1,OAS3,PGK1,RNF6,RSAD2,SEP15 | 11 |
| Neurological Disease | multiple sclerosis | 4.14E-05 | Increased | 2.433 | APP,IFIT1,IRF7,MX1,OAS1,OAS3,RSAD2 | 7 |
| Skeletal and Muscular Disorders | relapsing-remitting multiple sclerosis | 1.42E-08 | Increased | 2.433 | IFIT1,IRF7,MX1,OAS1,OAS3,RSAD2 | 6 |
| Skeletal and Muscular Disorders | neuromuscular disease | 5.95E-06 | Increased | 2.433 | APP,ATP2A2,FGF2,H3F3A/H3F3B,IFIT1,IRF7,MRPL9,MX1,OAS1,OAS3,PGK1,PPM1B,PPP1CB,PRNP,RARRES3,RSAD2,SACS,SEP15 | 18 |
| Skeletal and Muscular Disorders | multiple sclerosis | 4.14E-05 | Increased | 2.433 | APP,IFIT1,IRF7,MX1,OAS1,OAS3,RSAD2 | 7 |
| Vitamin and Mineral Metabolism | concentration of Ca2+ | 4.04E-04 | Increased | 2.200 | APP,ATP2A2,CXCL12,FGF2,RAB4A | 5 |
