# Supplementary material for: Reactive astrogliosis in response to hemorrhagic fever virus: microarray profile of Junin virus-infected human astrocytes
Source: Virol J. 2014 Jul 11;11:126. doi: 10.1186/1743-422X-11-126 (PMC4113780; doi:10.1186/1743-422X-11-126)
Supplement: Additional file 3: Table S3 — Predicted increase or decrease of biological functions in NHA in response to JUNV infection at 96 h p.i. Significance of Downstream Effects analysis was based on combination of the activation z-score (-2 ≥ Z ≥ 2) and overlap P-value (P < 0.05). Cell death functions are highlighted in grey. [file 1743-422X-11-126-S3.docx]

| **Category** | **Functions annotation** | **p-Value** | **Predicted activation state** | **Activation z-score** | **Molecules** | **Number of molecules** |
| --- | --- | --- | --- | --- | --- | --- |
| Cancer | infection of tumor cell lines | 2.74E-03 | Increased | 2.165 | ARGLU1,CD55,COPA,DDX58,DMXL1,EGFR,IFI35,IFITM1,MAT2A,MDM2,MED17,MED7,NMT1,RNF170,SEPT8,SIK1,SPATS2L,SPTAN1,SRSF2,ST3GAL5,WASF2,ZNF512B | 22 |
| Cancer | lymphohematopoietic cancer | 9.87E-06 | Decreased | -2.130 | ANXA4,ARID5B,BST2,BTG1,CD55,CFLAR,CRYAB,DDX58,DLC1,EGFR,EGR1,F11R,FADD,FBXL2,FUS,FYN,GSK3B,GTF2I,HDAC5,ID1,ID2,IDH2,LDHA,MDM2,MICA,MYD88,NQO1,PIM1,PRDM2,PSMA2,SRSF2,TFDP1,TGFBR2,TNFSF10,TRAF3,TUBB2A,TUBB4B,TUBB6,VEGFA,XRCC4,ZFP36 | 41 |
| Carbohydrate Metabolism | synthesis of carbohydrate | 3.46E-03 | Increased | 2.139 | BDKRB2,CHST15,CTGF,DUSP6,GNPDA1,GRK5,GSK3B,HRH1,IKBKB,MYD88,NGFR,PIGC,PIGL,PLSCR1,PPP1R15A,RGS3,SCD,SORBS1 | 18 |
| Cardiovascular Disease | hypertrophy of heart cells | 1.58E-03 | Increased | 2.582 | CRYAB,CTGF,DUSP1,GSK3B,HDAC5,IKBKB,MDM2,PIM1,RRAD | 9 |
| Cardiovascular Disease | hypertrophy of cardiomyocytes | 1.64E-03 | Increased | 2.582 | CRYAB,CTGF,DUSP1,GSK3B,HDAC5,IKBKB,MDM2,RRAD | 8 |
| Cell Death and Survival | apoptosis of leukocytes | 3.10E-03 | Increased | 2.312 | BTG1,CASP1,CASP6,CFLAR,CHI3L1,DDIT3,EGR1,EZR,FADD,FYN,GSK3B,IKBKB,MDM2,MYD88,NQO1,OGT,PIM1,TNFSF10,VEGFA | 19 |
| Cell Death and Survival | apoptosis of vascular endothelial cells | 1.21E-03 | Increased | 2.204 | ADM,CASP1,DDIT3,DUSP6,GSK3B,TNFSF10,VEGFA | 7 |
| Cell Death and Survival | apoptosis of lymphocytes | 4.31E-03 | Increased | 2.169 | CASP1,CASP6,CFLAR,CHI3L1,EGR1,EZR,FADD,FYN,GSK3B,IKBKB,MDM2,NQO1,OGT,TNFSF10,VEGFA | 15 |
| Cell Death and Survival | cell death of cardiomyocytes | 1.98E-03 | Increased | 2.119 | ADM,CASP1,CRYAB,FADD,GSK3B,MDM2,PIM1,PPP1R15A,RRAD,TFDP1,VEGFA | 11 |
| Cell Death and Survival | apoptosis of T lymphocytes | 1.42E-03 | Increased | 2.082 | CASP1,CASP6,CFLAR,CHI3L1,EGR1,EZR,FADD,FYN,GSK3B,IKBKB,MDM2,NQO1,OGT,TNFSF10 | 14 |
| Cell Death and Survival | apoptosis of cardiomyocytes | 1.99E-03 | Increased | 2.077 | ADM,CASP1,CRYAB,FADD,GSK3B,MDM2,PIM1,RRAD,TFDP1,VEGFA | 10 |
| Cell Death and Survival | apoptosis of blood cells | 3.30E-03 | Increased | 2.054 | BTG1,CASP1,CASP6,CFLAR,CHI3L1,DDIT3,EGR1,EZR,FADD,FYN,GSK3B,IKBKB,MDM2,MYD88,NQO1,OGT,PIM1,TNFSF10,TRAF3,VEGFA | 20 |
| Cellular Development | differentiation of leukocytes | 1.25E-03 | Increased | 2.254 | DDX58,EGR1,EGR2,ELF4,FYN,GSK3B,HDAC5,HRH1,ID1,ID2,MGAT2,MLLT11,MYD88,NCOA6,NRP1,OGT,PLSCR1,RSAD2,SPEN,TGFBR2,TNFRSF11B,TNFSF10,TRAF3,VEGFA,XRCC4,ZFP36 | 26 |
| Cellular Development | differentiation of blood cells | 1.67E-03 | Increased | 2.182 | DDX58,EGFR,EGR1,EGR2,ELF4,FADD,FYN,GSK3B,HDAC5,HRH1,ID1,ID2,MGAT2,MLLT11,MYD88,NCOA6,NRP1,OGT,PLSCR1,RSAD2,SPEN,TGFBR2,TNFRSF11B,TNFSF10,TRAF3,VEGFA,WASF2,XRCC4,ZFP36 | 29 |
| Cellular Development | proliferation of gonadal cell lines | 3.48E-04 | Decreased | -2.000 | BDKRB2,CSNK2A1,EGFR,EGR2,PLSCR1,RARRES3,TUBB4B | 7 |
| Cellular Growth and Proliferation | proliferation of gonadal cell lines | 3.48E-04 | Decreased | -2.000 | BDKRB2,CSNK2A1,EGFR,EGR2,PLSCR1,RARRES3,TUBB4B | 7 |
| Cellular Movement | infiltration by neutrophils | 2.95E-04 | Increased | 2.047 | ADM,C3,CASP1,CTGF,DUSP1,EGFR,IKBKB,MYD88,NQO1,TGFBR2 | 10 |
| Developmental Disorder | hypertrophy of heart cells | 1.58E-03 | Increased | 2.582 | CRYAB,CTGF,DUSP1,GSK3B,HDAC5,IKBKB,MDM2,PIM1,RRAD | 9 |
| Developmental Disorder | hypertrophy of cardiomyocytes | 1.64E-03 | Increased | 2.582 | CRYAB,CTGF,DUSP1,GSK3B,HDAC5,IKBKB,MDM2,RRAD | 8 |
| DNA Replication, Recombination, and Repair | degradation of DNA | 1.78E-03 | Increased | 2.734 | ADM,CASP6,CRYAB,DDIT3,DUSP1,FADD,GSK3B,HNRNPA1,ISG20,TIAM1,TNFSF10 | 11 |
| Gene Expression | transactivation | 1.87E-04 | Increased | 2.917 | CTGF,DDIT3,DDX58,EGR1,EGR2,FUS,GLI2,GTF2F1,GTF2I,HNRNPA1,ID1,IKBKB,IRF9,KHDRBS1,MDM2,MED17,MED7,MX1,MYD88,NCOA6,NDN,PER1,PIM1,PRMT2,SRSF2,TFDP1,TNFSF10 | 27 |
| Gene Expression | expression of RNA | 4.53E-06 | Increased | 2.697 | ANG,APBB2,ARID5B,BDKRB2,BST2,BTG1,CASP1,CDKN1C,CFLAR,CTGF,CTR9,DDIT3,DDX58,DIRAS3,DNAJB6,DUSP1,EGFR,EGR1,EGR2,EIF2B1,EIF4B,ELF4,FADD,GLI2,GSK3B,GTF2F1,GTF2I,GTF2IRD1,HDAC5,HNRNPA1,ID1,ID2,IKBKB,IRF9,ISG20,KHDRBS1,LITAF,MDM2,MED17,MED7,MKL2,MLLT11,MYD88,NCOA6,NDN,NPAS2,NQO1,OGT,PABPC4,PABPN1,PER1,PKNOX2,PLSCR1,PRDM2,PRMT2,RARRES3,RFX5,RUVBL1,SATB2,SFPQ,SGMS1,SIK1,SOX9,SPEN,SQSTM1,TARDBP,TFDP1,TGFBR2,TLE4,TNFSF10,TRADD,TRAF3,VEGFA,ZFP36,ZNF274,ZNHIT3 | 76 |
| Gene Expression | transactivation of RNA | 2.71E-04 | Increased | 2.664 | DDIT3,DDX58,EGR1,EGR2,FUS,GLI2,GTF2F1,GTF2I,HNRNPA1,ID1,IKBKB,IRF9,KHDRBS1,MDM2,MED17,MED7,MX1,MYD88,NCOA6,NDN,PER1,PIM1,PRMT2,SRSF2,TFDP1,TNFSF10 | 26 |
| Gene Expression | transcription of RNA | 4.21E-06 | Increased | 2.399 | ANG,APBB2,ARID5B,BDKRB2,BST2,BTG1,CASP1,CDKN1C,CFLAR,CTGF,CTR9,DDIT3,DDX58,DNAJB6,DUSP1,EGFR,EGR1,EGR2,ELF4,FADD,GLI2,GSK3B,GTF2F1,GTF2I,GTF2IRD1,HDAC5,HNRNPA1,ID1,ID2,IKBKB,IRF9,KHDRBS1,LITAF,MDM2,MED17,MED7,MKL2,MLLT11,MYD88,NCOA6,NDN,NPAS2,OGT,PABPN1,PER1,PKNOX2,PLSCR1,PRDM2,PRMT2,RARRES3,RFX5,RUVBL1,SATB2,SFPQ,SIK1,SOX9,SPEN,SQSTM1,TARDBP,TFDP1,TGFBR2,TLE4,TNFSF10,TRADD,TRAF3,VEGFA,ZFP36,ZNF274,ZNHIT3 | 69 |
| Gene Expression | transcription | 4.16E-06 | Increased | 2.383 | ANG,APBB2,ARID5B,BDKRB2,BST2,BTG1,CASP1,CDKN1C,CFLAR,CTGF,CTR9,DDIT3,DDX58,DNAJB6,DUSP1,EGFR,EGR1,EGR2,ELF4,FADD,GLI2,GSK3B,GTF2F1,GTF2I,GTF2IRD1,HDAC5,HNRNPA1,ID1,ID2,IKBKB,IRF9,KHDRBS1,LITAF,MDM2,MED17,MED7,MKL2,MLLT11,MX1,MYD88,NCOA6,NDN,NPAS2,OGT,PABPN1,PER1,PKNOX2,PLSCR1,PRDM2,PRMT2,RARRES3,RFX5,RUVBL1,SATB2,SFPQ,SIK1,SOX9,SPEN,SQSTM1,TARDBP,TFDP1,TGFBR2,TLE4,TNFSF10,TRADD,TRAF3,VEGFA,ZFP36,ZNF274,ZNHIT3 | 70 |
| Hematological System Development and Function | differentiation of leukocytes | 1.25E-03 | Increased | 2.254 | DDX58,EGR1,EGR2,ELF4,FYN,GSK3B,HDAC5,HRH1,ID1,ID2,MGAT2,MLLT11,MYD88,NCOA6,NRP1,OGT,PLSCR1,RSAD2,SPEN,TGFBR2,TNFRSF11B,TNFSF10,TRAF3,VEGFA,XRCC4,ZFP36 | 26 |
| Hematological System Development and Function | differentiation of blood cells | 1.67E-03 | Increased | 2.182 | DDX58,EGFR,EGR1,EGR2,ELF4,FADD,FYN,GSK3B,HDAC5,HRH1,ID1,ID2,MGAT2,MLLT11,MYD88,NCOA6,NRP1,OGT,PLSCR1,RSAD2,SPEN,TGFBR2,TNFRSF11B,TNFSF10,TRAF3,VEGFA,WASF2,XRCC4,ZFP36 | 29 |
| Hematological System Development and Function | infiltration by neutrophils | 2.95E-04 | Increased | 2.047 | ADM,C3,CASP1,CTGF,DUSP1,EGFR,IKBKB,MYD88,NQO1,TGFBR2 | 10 |
| Hematopoiesis | differentiation of leukocytes | 1.25E-03 | Increased | 2.254 | DDX58,EGR1,EGR2,ELF4,FYN,GSK3B,HDAC5,HRH1,ID1,ID2,MGAT2,MLLT11,MYD88,NCOA6,NRP1,OGT,PLSCR1,RSAD2,SPEN,TGFBR2,TNFRSF11B,TNFSF10,TRAF3,VEGFA,XRCC4,ZFP36 | 26 |
| Immune Cell Trafficking | infiltration by neutrophils | 2.95E-04 | Increased | 2.047 | ADM,C3,CASP1,CTGF,DUSP1,EGFR,IKBKB,MYD88,NQO1,TGFBR2 | 10 |
| Infectious Disease | infection by lentivirus | 1.23E-03 | Increased | 3.214 | ARGLU1,C1R,C1S,C3,C6orf48,CFI,CRYAB,DLGAP4,DMXL1,EGFR,HCP5,KHDRBS1,MAT2A,MDM2,MED17,MED7,NCOA6,NMT1,PSMA2,RNF170,SEPT8,SIK1,SPATS2L,SPEN,SPG7,SPTAN1,SRSF2,ST3GAL5,ZNF512B | 29 |
| Infectious Disease | HIV infection | 2.36E-03 | Increased | 3.095 | ARGLU1,C1R,C1S,C3,C6orf48,CFI,CRYAB,DLGAP4,DMXL1,EGFR,HCP5,KHDRBS1,MAT2A,MED17,MED7,NCOA6,NMT1,PSMA2,RNF170,SEPT8,SIK1,SPATS2L,SPEN,SPG7,SPTAN1,SRSF2,ST3GAL5,ZNF512B | 28 |
| Infectious Disease | infection of tumor cell lines | 2.74E-03 | Increased | 2.165 | ARGLU1,CD55,COPA,DDX58,DMXL1,EGFR,IFI35,IFITM1,MAT2A,MDM2,MED17,MED7,NMT1,RNF170,SEPT8,SIK1,SPATS2L,SPTAN1,SRSF2,ST3GAL5,WASF2,ZNF512B | 22 |
| Infectious Disease | replication of Murine herpesvirus 4 | 6.51E-06 | Decreased | -2.236 | DDX58,ISG20,MX2,OAS1,PARP12 | 5 |
| Infectious Disease | replication of Hepatitis C virus | 1.54E-04 | Decreased | -2.479 | DDX58,IFI27,IFI6,IFIT1,IFITM1,ISG15,RSAD2 | 7 |
| Infectious Disease | replication of virus | 3.17E-07 | Decreased | -2.665 | BST2,COPA,DDX58,EGR1,F11R,GRK5,GSK3B,IFI27,IFI6,IFIT1,IFITM1,IRF9,ISG15,ISG20,MDM2,MX1,MX2,MYD88,OAS1,OASL,PANX1,PARP12,PPP1R15A,PRDM2,RARRES3,RSAD2,SFPQ,SRSF1,TGFBR2,TNFSF10,TRADD,TRIM14 | 32 |
| Infectious Disease | replication of RNA virus | 1.25E-06 | Decreased | -2.708 | BST2,COPA,DDX58,F11R,GRK5,GSK3B,IFI27,IFI6,IFIT1,IFITM1,IRF9,ISG15,ISG20,MDM2,MX1,MYD88,OAS1,OASL,PANX1,PARP12,PPP1R15A,PRDM2,RARRES3,RSAD2,SFPQ,SRSF1,TNFSF10,TRADD,TRIM14 | 29 |
| Infectious Disease | replication of vesicular stomatitis virus | 1.37E-05 | Decreased | -2.717 | DDX58,IRF9,ISG20,OAS1,OASL,PARP12,RARRES3,TRADD | 8 |
| Inflammatory Disease | relapsing-remitting multiple sclerosis | 1.54E-06 | Increased | 2.630 | IFIT1,ISG15,MX1,OAS1,OAS3,RSAD2,SERPING1 | 7 |
| Inflammatory Disease | multiple sclerosis | 2.72E-05 | Increased | 2.630 | CASP1,CRYAB,DDIT3,EGR1,IFIT1,ISG15,KCNMA1,MX1,NGFR,OAS1,OAS3,RSAD2,SERPING1 | 13 |
| Inflammatory Response | infiltration by neutrophils | 2.95E-04 | Increased | 2.047 | ADM,C3,CASP1,CTGF,DUSP1,EGFR,IKBKB,MYD88,NQO1,TGFBR2 | 10 |
| Lipid Metabolism | synthesis of lipid | 1.31E-03 | Increased | 2.943 | ADM,ANG,BDKRB2,C3,CASP1,DOLK,EGFR,EGR1,FYN,HRH1,INSIG1,MGAT2,MYD88,NAA40,NGFR,PEX11B,PIGC,PIGL,PLSCR1,PTGIS,RGS3,SCD,SGMS1,SORBS1,SPTSSA,ST3GAL5,TNFSF10,VEGFA | 28 |
| Neurological Disease | neuromuscular disease | 3.35E-06 | Increased | 3.098 | C3,CASP1,CASP6,CDKN1C,CFLAR,CHI3L1,CRYAB,CTGF,DDIT3,DNAJB6,EGR1,EGR2,F11R,GSK3B,HDAC5,HRH1,IFIT1,IKBKB,ISG15,KCNAB1,KCNMA1,LDHA,MX1,NGFR,NREP,OAS1,OAS3,PCDH7,PRDM2,RARRES3,RNF114,RSAD2,SACS,SCD,SERPING1,SLC25A6,SOX9,SRSF1,SRSF2,ST3GAL5,ZMYND8 | 41 |
| Neurological Disease | progressive motor neuropathy | 2.27E-06 | Increased | 2.813 | ABAT,ANG,CASP1,CRYAB,DDIT3,DNAJB6,EGR1,FADD,FUS,GSK3B,HRH1,IFIT1,ISG15,KCNMA1,LDHA,MX1,NGFR,OAS1,OAS3,PRDM2,RNF114,RSAD2,SERPING1,SLC25A6,TARDBP | 25 |
| Neurological Disease | relapsing-remitting multiple sclerosis | 1.54E-06 | Increased | 2.630 | IFIT1,ISG15,MX1,OAS1,OAS3,RSAD2,SERPING1 | 7 |
| Neurological Disease | multiple sclerosis | 2.72E-05 | Increased | 2.630 | CASP1,CRYAB,DDIT3,EGR1,IFIT1,ISG15,KCNMA1,MX1,NGFR,OAS1,OAS3,RSAD2,SERPING1 | 13 |
| Organismal Survival | organismal death | 4.55E-03 | Decreased | -3.095 | ADM,APBB2,ARID5B,BDKRB2,C3,CASP1,CDKN1C,CELF1,CFLAR,CRYAB,CSNK2A1,CTGF,CTTN,DDX58,DLC1,DNAJB6,DUSP1,EGFR,EGR2,FADD,FYN,FZR1,GCLM,GLI2,GSK3B,HDAC5,HEG1,HRH1,ID1,ID2,IKBKB,INSIG1,IRF9,ISG15,KCNAB1,KCNMA1,KHDRBS1,MDM2,MGAT2,MKL2,MYD88,NCOA6,NDN,NGFR,NQO1,NRP1,PAFAH1B1,PEX11B,PPP1R15A,PRDM2,SATB2,SERPING1,SGMS1,SIK3,SLC25A13,SOX9,SPEN,SPRY1,SQSTM1,SUN1,TARDBP,TBC1D4,TFDP1,TGFBR2,TNFRSF11B,TNFSF10,TRAF3,USP18,VEGFA,WASF2,XRCC4,ZFP36 | 72 |
| Reproductive System Development and Function | proliferation of gonadal cell lines | 3.48E-04 | Decreased | -2.000 | BDKRB2,CSNK2A1,EGFR,EGR2,PLSCR1,RARRES3,TUBB4B | 7 |
| Skeletal and Muscular Disorders | neuromuscular disease | 3.35E-06 | Increased | 3.098 | C3,CASP1,CASP6,CDKN1C,CFLAR,CHI3L1,CRYAB,CTGF,DDIT3,DNAJB6,EGR1,EGR2,F11R,GSK3B,HDAC5,HRH1,IFIT1,IKBKB,ISG15,KCNAB1,KCNMA1,LDHA,MX1,NGFR,NREP,OAS1,OAS3,PCDH7,PRDM2,RARRES3,RNF114,RSAD2,SACS,SCD,SERPING1,SLC25A6,SOX9,SRSF1,SRSF2,ST3GAL5,ZMYND8 | 41 |
| Skeletal and Muscular Disorders | relapsing-remitting multiple sclerosis | 1.54E-06 | Increased | 2.630 | IFIT1,ISG15,MX1,OAS1,OAS3,RSAD2,SERPING1 | 7 |
| Skeletal and Muscular Disorders | multiple sclerosis | 2.72E-05 | Increased | 2.630 | CASP1,CRYAB,DDIT3,EGR1,IFIT1,ISG15,KCNMA1,MX1,NGFR,OAS1,OAS3,RSAD2,SERPING1 | 13 |
| Skeletal and Muscular Disorders | hypertrophy of cardiomyocytes | 1.64E-03 | Increased | 2.582 | CRYAB,CTGF,DUSP1,GSK3B,HDAC5,IKBKB,MDM2,RRAD | 8 |
| Small Molecule Biochemistry | synthesis of lipid | 1.31E-03 | Increased | 2.943 | ADM,ANG,BDKRB2,C3,CASP1,DOLK,EGFR,EGR1,FYN,HRH1,INSIG1,MGAT2,MYD88,NAA40,NGFR,PEX11B,PIGC,PIGL,PLSCR1,PTGIS,RGS3,SCD,SGMS1,SORBS1,SPTSSA,ST3GAL5,TNFSF10,VEGFA | 28 |
