# Supplementary material for: Reactive astrogliosis in response to hemorrhagic fever virus: microarray profile of Junin virus-infected human astrocytes
Source: Virol J. 2014 Jul 11;11:126. doi: 10.1186/1743-422X-11-126 (PMC4113780; doi:10.1186/1743-422X-11-126)
Supplement: Additional file 4: Table S4 — High correlation of microarray and qRT-PCR data for a subset of immune response genes. Confirmation of expression of selected differentially expressed (array) IFN signaling genes at 24 and 96 h post JUNV infection of NHA by qRT-PCR using cDNA from JUNV-infected NHA and from mock-infected control cells. P-values originated from statistical analysis of fold-changes for qRT-PCR are provided in the table. ND, not determined. [file 1743-422X-11-126-S4.docx]

| **Affymetrix ID** | **Entrez gene ID for human** | **Symbol** | **J24vsM24** | | | **J96vsM96** | | |
| --- | --- | --- | --- | --- | --- | --- | --- | --- |
|  |  |  | **Log2(fold change)** | | **qRT-PCR *P*-value** | **Log2(fold change)** | | **qRT-PCR *P*-value** |
|  |  |  | **Array** | **qRT-PCR** |  | **Array** | **qRT-PCR** |  |
| 218943_s_at | 23586 | DDX58 | 4.7 | 4.787876 | 0.007306 | 3.759 | 5.467934 | 0.004507 |
| 202411_at | 3429 | IFI27 | 2.488 | 5.056024 | 0.000061 | 9.585 | 11.6999 | 0.000007 |
| 209417_s_at | 3430 | IFI35 |  | ND |  | 4.839 | 4.363315 | 0.001475 |
| 214453_s_at | 10561 | IFI44 |  | ND |  | 4.569 | 4.497535 | 0.004725 |
| 204439_at | 10964 | IFI44L | 6.73 | 6.798029 | 0.000191 | 7.474 | 7.58962 | 0.000191 |
| 204415_at | 2537 | IFI6 | 5.716 | 5.23034 | 0.000025 | 6.645 | 9.10491 | 0.000094 |
| 203153_at | 3434 | IFIT1 | 9.244 | 7.841946 | 0.000903 | 7.418 | 6.550561 | 0.010381 |
| 204747_at | 3437 | IFIT3 | 4.87 | 5.469232 | 0.001573 |  | ND |  |
| 201601_x_at | 8519 | IFITM1 | 4.767 | 5.847527 | 0.000222 | 6.312 | 8.570798 | 0.000014 |
| 214022_s_at |  |  | 6.32 |  |  | 6.47 |  |  |
| 208436_s_at | 3665 | IRF7 | 3.065 | 0.978459 | 0.013551 |  | ND |  |
| 203882_at | 10379 | IRF9 |  | ND |  | 3.492 | 2.220127 | 0.000348 |
| 205483_s_at | 9636 | ISG15 |  | ND |  | 5.317 | 6.286759 | 0.000134 |
| 204698_at | 3669 | ISG20 |  | ND |  | 3.489 | 4.509596 | 0.00219 |
| 202086_at | 4599 | MX1 | 7.295 | 0.805436 | 0.038599 | 7.211 | 0.937365 | 0.00485 |
| 204994_at | 4600 | MX2 | 5.407 | 7.896216 | 0.000001 | 5.675 | 8.906223 | 0.000031 |
| 209124_at | 4615 | MYD88 |  | ND |  | 3.774 | 3.591723 | 0.000799 |
| 205552_s_at | 4938 | OAS1 | 5.259 | 8.085445 | 0.000041 | 7.746 | 8.07365 | 0.000154 |
| 202869_at |  |  | 5.212 |  |  | 5.648 |  |  |
| 204972_at | 4939 | OAS2 | 3.223 | 7.235248 | 0.000144 | 6.451 | 9.175611 | 0.000011 |
| 218400_at | 4940 | OAS3 | 4.828 | 2.991916 | 0.000033 | 6.837 | 4.329144 | 0.000134 |
| 205660_at | 8638 | OASL | 6.529 | 9.063141 | 0.000006 | 6.167 | 8.67328 | 0.000003 |
| 210797_s_at |  | OASL | 4.888 |  |  | 5.264 |  |  |
